# Virome Assembly and Annotation: A Surprise in the Namib Desert

**DOI:** 10.3389/fmicb.2017.00013

**Published:** 2017-01-23

**Authors:** Uljana Hesse, Peter van Heusden, Bronwyn M. Kirby, Israel Olonade, Leonardo J. van Zyl, Marla Trindade

**Affiliations:** ^1^Institute for Microbial Biotechnology and Metagenomics, University of the Western CapeBellville, South Africa; ^2^South African National Bioinformatics Institute, University of the Western CapeBellville, South Africa

**Keywords:** Namib Desert, virome, circovirus, assembly, annotation, simulated/virtual metagenomes

## Abstract

Sequencing, assembly, and annotation of environmental virome samples is challenging. Methodological biases and differences in species abundance result in fragmentary read coverage; sequence reconstruction is further complicated by the mosaic nature of viral genomes. In this paper, we focus on biocomputational aspects of virome analysis, emphasizing latent pitfalls in sequence annotation. Using simulated viromes that mimic environmental data challenges we assessed the performance of five assemblers (CLC-Workbench, IDBA-UD, SPAdes, RayMeta, ABySS). Individual analyses of relevant scaffold length fractions revealed shortcomings of some programs in reconstruction of viral genomes with excessive read coverage (IDBA-UD, RayMeta), and in accurate assembly of scaffolds ≥50 kb (SPAdes, RayMeta, ABySS). The CLC-Workbench assembler performed best in terms of genome recovery (including highly covered genomes) and correct reconstruction of large scaffolds; and was used to assemble a virome from a copper rich site in the Namib Desert. We found that scaffold network analysis and cluster-specific read reassembly improved reconstruction of sequences with excessive read coverage, and that strict data filtering for non-viral sequences prior to downstream analyses was essential. In this study we describe novel viral genomes identified in the Namib Desert copper site virome. Taxonomic affiliations of diverse proteins in the dataset and phylogenetic analyses of circovirus-like proteins indicated links to the marine habitat. Considering additional evidence from this dataset we hypothesize that viruses may have been carried from the Atlantic Ocean into the Namib Desert by fog and wind, highlighting the impact of the extended environment on an investigated niche in metagenome studies.

## Introduction

Viruses are important players in their respective ecological niches. Not only do they drive host adaptation through horizontal gene transfer, they also regulate microbial abundance and diversity through predation (reviewed in Kimura et al., 2008). The latter effect is so extensive that they are now recognized as a major driving force in global biogeochemical nutrient cycles (Fuhrman, 1999). The advances in next generation sequencing technologies have boosted studies on viral diversity and functionality through metagenome and virome analysis. An increasing number of studies investigate virome data obtained from purified viral particles, following a workflow similar to the one established for marine viromes (Solonenko and Sullivan, 2013). In short, it involves filtering and concentration of viral particles from the environmental sample, DNAse, and RNAse treatments to reduce genomic contamination from non-viral organisms, degradation of the protein coats, purification of the DNA, and sequencing of the viral genomes. To date, few studies have used this approach to investigate viromes derived from soils (reviewed in Zablocki et al., 2016). This medium is challenging, as it is rich in enzyme-inhibiting compounds that interfere with library construction (Fenner and Freeman, 2011; Carvalhais et al., 2012; Tveit et al., 2014). To minimize the effects of such inhibitors, additional purification steps, and whole genome amplification (WGA) are sometimes unavoidable (e.g., Zablocki et al., 2014; Adriaenssens et al., 2016). Yet, such methods can introduce biases, such as DNA fragmentation and selective amplification of DNA (Kim and Bae, 2011; Yuan et al., 2012; van Dijk et al., 2014), which are then reflected in the read datasets.

Assembly of reads into longer sequences facilitates identification of coding genes, as well as taxonomic and functional annotation. However, correct reassembly of virome data is challenging. Highly variable read coverage between and across the diverse viral genomes may lead to missing sequence information and fragmented genome recovery (García-López et al., 2015). The mosaic structure of viral genomes caused by recurrent horizontal exchange of genetic material (reviewed in Hatfull, 2008) further complicates the matter. Several metagenome assemblers that account for varying sequencing depths in the data have been developed (e.g., Peng et al., 2011; Bankevich et al., 2012; Boisvert et al., 2012). Their suitability for virome assembly has been assessed in recent studies (de Cárcer et al., 2014; Vázquez-Castellanos et al., 2014; García-López et al., 2015; Laffy et al., 2016). This was effectively done using simulated data: virtual viromes consisting of known viral genomes at varying abundance levels serve to generate synthetic reads, which are reassembled into contigs and scaffolds. The reassembled sequences can then be mapped back to the original viral genomes, providing information on assembly precision. Based on these studies, the programs IDBA-UD, CLC, RayMeta, and SPAdes produced the most accurate assemblies. However, these assemblers have not been tested for their ability to correctly reconstruct fragmented DNA and genomic sequences with excessive read coverage, as may occur during DNA purification procedures and WGA.

Currently, virome annotation is often conducted using only the viral subset of the RefSeq database (e.g., Minot et al., 2013; Carlos et al., 2014; Weynberg et al., 2014; Zablocki et al., 2014; Hannigan et al., 2015; Mohiuddin and Schellhorn, 2015). This can be achieved through direct BLAST analyses or using the convenient and informative online annotation server MetaVir (Roux et al., 2011). Other programs for virome annotation include VMGAP (Lorenzi et al., 2011), VIROME (Wommack et al., 2012), and MG-Rast (Meyer et al., 2008); however, they have been found less effective in identification of viral strains and taxa as compared to MetaVir or direct BLAST analysis (Tangherlini et al., 2016). One major drawback of this approach is that it does not discriminate between viral and non-viral DNA: any contaminating non-viral sequence that shows significant similarity to a viral gene will be annotated as viral. MetaVir does not filter for non-viral sequences, but relies on the user to perform such analyses. Diverse methods (e.g., filtering for 16S rRNA and tRNA) and tools (e.g., Schmieder and Edwards, 2011; Roux et al., 2015) for prescreening virome data have been applied. The recently published virome annotation program HoloVir (Laffy et al., 2016), addresses this problem by prescreening the sequences against the entire SILVA database and an extensive database with viral and cellular marker genes. However, comparative analyses assessing the efficiency of these procedures are still outstanding.

Despite severe environmental constraints, deserts host a multitude of animals, plants, and microorganisms, and are estimated to store almost one third of the global terrestrial carbon (Pointing and Belnap, 2012; Makhalanyane, 2015). Virus research in hot desert soils using classical techniques (Prigent et al., 2005; Prestel et al., 2008, 2012) and metagenomic approaches (Fierer et al., 2007; Adriaenssens et al., 2015; Vikram et al., 2015; Adriaenssens et al., 2016), revealed a wide diversity of viral taxa with a prevalence of tailed viruses (Caudovirales), and described a range of novel viral proteins. Yet, the extent of extracellular viral diversity and ecological functions of viruses in these arid ecosystems remain severely understudied. The Namib Desert is described as one of the oldest deserts in the world, deemed semi-arid for an estimated 55–80 million years (Goudie, 2010). Large areas are currently considered hyper-arid with scant, erratic and low precipitation inputs. Surface temperatures are subject to extreme seasonal fluctuation, reaching up to 70°C for short periods of time. High levels of UV radiation, physical disturbance and low nutrient status further accentuate the severe conditions (Nicholson, 2011; Eckardt et al., 2013b). The surface geomorphology and mineralogy in the Namib Desert also varies widely, with reported surface outcrops of uranium and copper (Eckardt et al., 2013b). These create environmental pockets where microorganisms and their respective viral pathogens may have adapted to poly-extreme conditions. Studies have shown that ore microbes attach to ore particles, exhibiting a general tolerance to high concentrations of metallic and other ions (Dopson et al., 2003; Rawlings, 2005). The dynamic, poly-extreme conditions of desert ore sites make them particularly attractive environments for studies on viral ecology and evolution. Viral communities associated with desert ores have not been assessed before, and their adaptation to such an environment could be exploited in numerous ways for biotechnological application.

In this paper we investigate viral diversity at a copper deposit in the Namib Desert and describe novel viral genomes. Furthermore, we assess effectiveness and accuracy of five assemblers using simulated datasets that mimic environmental data challenges (DNA fractionation and excessive coverage of selected genomes), and address presence of non-viral DNA in the copper soil virome and other datasets.

## Materials and methods

### Simulated virome data analyses

Initial assembly tests of the Namib Desert copper site data indicated excessive read coverage for small circovirus-like genomes (~2 kb) and fragmental representation of larger viral genomes. To investigate which assembly program excels in accuracy and genome recovery given such data, we generated three virtual viromes (Sim1, Sim2, and Sim3). Sim1 consisted of 572 complete viral genomes at relative abundances as published by Vázquez-Castellanos et al. (2014). Sim2 (testing fractionation effects) consisted of non-overlapping 2 kb sequences from these viral genomes, and Sim3 (testing excessive read coverage for small circovirus-like genomes) consisted of the above 572 complete viral genomes at original abundance levels plus five published circoviral genomes with exceptionally high abundance levels. The simulated read datasets were produced using MetaSim (Richter et al., 2008). Since a 250 nt error model was not available, we generated datasets of read length 80 nt (errormodel-80bp.mconf; MetaSim homepage) and read length 300 nt (errormodel-300nt.mconf; http://seqanswers.com/forums/showthread.php?t=44676). Using the empirical error model and an insert size of 450 nt (10% std), we produced 4.5 Mio read pairs with Illumina-specific errors (error rate 0.9 and 1% for 80 and 300 nt, respectively) for each of the six datasets, respectively.

The six virome datasets were assembled using five assemblers. IDBA-UD (Peng et al., 2012) and SPAdes (Bankevich et al., 2012) conduct assembly iterating through several kmers. With these assemblers, default settings were used with the 80 nt read datasets. With the 300 nt datasets additional kmers (IDBA-UD: kmer 40, 80, 120; SPAdes: default, 79, 119) were used. Assemblies with the CLC-Genomic-Workbench (version 7.5, http://www.clcbio.com) were conducted using automated kmer length (resulting in kmer 23), and specifying 95% read coverage and 95% nucleotide identity for read mapping. ABySS (Simpson et al., 2009) and RayMeta (Boisvert et al., 2012) were run with default assembly parameters and a kmer length of 23.

Assembly quality was assessed by (1) aligning all contigs and scaffolds larger than 500 nt from the simulated datasets against their respective genomes using MUMmer v-3 (Kurtz et al., 2004), (2) mapping all reads back to the assembled contigs using Bowtie2 v-2.2.6 (Langmead and Salzberg, 2012), selecting end-to-end matches (–no-1 mm-upfront switched on) and reporting multiple alignments (-k 3); and (3) running ALE (Clark et al., 2013) on all assembled datasets to compare the performance of the different assemblers. Genomes were considered “recovered” if they were reassembled into one sequence.

### Virus isolation, concentration, DNA extraction, and sequencing

This study concentrates on a specific environmental niche: isolated heaps of copper ore material found in the vicinity of former copper mines in the Namib Desert. Sample processing was conducted similar to previously published methodologies (Adriaenssens et al., 2015). Two 25 L carboys were each half filled with rocks and soil from the sample site (4 × 2.5 m; 23°33′35.27″S; 15°16′50.63″E; Figure S1), then completely filled with distilled water and shaken for 20 min. The resulting slurry was left to settle for an hour and the supernatant was decanted into a clean carboy. The drums with sediment were again filled with distilled water and shaken, left to settle for an hour, and the supernatant decanted into the same collection carboy. The supernatant was filtered through a Millipore 1 μm Polygard-CR filter (CR0101006) using a Millipore peristaltic pump (XX80EL230) and the filtrate collected in a clean carboy. The 1 μm filtrate was filtered through a 0.22 μm Opticap® XL10 Durapore® filter (KVGLA10HH1) and the filtrate collected in a clean carboy. The 0.22 μm filtrate was subjected to tangential flow filtration (TFF) using a Millipore TFF cartridge holder (XX42PS001) and 30 kDa cut-off filter (CDUF001TT). The ±50L of 0.22 μm filtrate was concentrated to ±140 ml (360 × concentrated) at the Gobabeb research center, and the viral fraction stored at 4°C for 2 weeks prior to DNA extraction in the laboratory at the University of the Western Cape. Virus and virus-like particles were collected for DNA extraction by centrifuging 50 ml of the concentrate at 25.000 × g for 6 h using a Beckman Avanti J-26 XPI centrifuge in a JA20 rotor. The pellet was resuspended in 200 μl TE buffer. This viral suspension was treated with DNaseI (EN0521) and RNaseA (EN0531) (Fermentas—final concentration of 0.1 μg/ml) at 37°C for 1 h. We tested for presence of bacterial DNA by amplifying the 16S rRNA gene using the primers E9F and U1510R (Hansen et al., 1998; Baker et al., 2003) as follows: 1 μl of genomic DNA was mixed with 2.5 μl of each primer (10 mM), 2.5 μl of 2 μM dNTPs, 2.5 μl of 10X DreamTaq buffer (ThermoFisher Scientific, MA, USA), 1 μl BSA 10 mg/ml, 0.125 μl DreamTaq polymerase (ThermoFisher Scientific, MA, USA) and milliQ water to a total volume of 25 μl. PCR was conducted under the following thermal regime: 95°C for 5 min, 95°C for 30 s, 52°C for 30 s, 72°C for 85 s (30 cycles), and 10 min at 72°C. The sample was deemed free of bacterial DNA when 16S rDNA could be amplified only from the positive controls, but not from the sample or the negative controls. Then, the virus particles were treated with Proteinase K (Fermentas—final concentration 1 μg/ml) at 55°C for 2 h. Thirty microliters of 20% SDS were added and the sample incubated at 37°C for 1 h. The DNA was extracted with three phenol:chloroform:isoamylalcohol (25:24:1) extractions, followed by two extractions using chloroform:isoamylalcohol (24:1). Phase separation was achieved by centrifugation in an Eppendorf 5810R centrifuge at 5000 RPM for 10 min. Precipitation of the DNA was performed through addition of 1/10 volume of 3 M NaOAc (pH 5.2) and 2 volumes 95% ethanol, with overnight incubation at 4°C. Precipitated DNA was recovered by centrifugation at 13,000 RPM for 10 min and the resulting pellet was resuspended in 30 μl of TE buffer. DNA was purified using the QIAamp DNA stool mini kit (cat. no. 51504) using half of an Inhibit EX tablet (provided with this kit) per purification. Then, 10 ng of the extracted, purified DNA was used to perform WGA (GE Healthcare GenomiPhi HY DNA amplification kit cat. no. 25-6600-20) using the manufacturer's recommendations. The resulting DNA was purified using the Qiagen Gel Extraction kit (Qiaex II, cat. no. 20021). Throughout the extraction and purification process the sample was assessed for the presence of polymerase inhibitors via PCR. Using the above primers, a 16S rRNA gene PCR reaction of genomic *E. coli* DNA was spiked with ~1 ng extracted metagenomic DNA and the level of amplification was compared to an unspiked control reaction containing only genomic DNA. The sequence library was prepared with the Illumina Nextera XT library prep kit with minor modifications. The amount of input DNA was decreased to 0.8 ng and 1U Phusion polymerase (Thermo Scientific, cat no. F-530S) was included in the tagmentation reaction. The amplified DNA was sequenced with a MiSeq Reagent V2 kit (2 × 250 nt reads) on the Illumina MiSeq Sequencing platform and included a 20% PhiX V3 spike as per manufacturer's instructions (Preparation guide, Part #15031942, May 2012 revision).

### Transmission electron microscopy of viruses and virus like particles

Virus particle suspensions were prepared according to the ammonium acetate method as described by Ackermann (2009). Three microliters taken from the 140 ml concentrate were pipetted onto carbon coated 200 mesh copper grids and stained with 2% aqueous uranyl acetate for 30 s. The samples were viewed using a LEO 912 Omega TEM (Zeiss, Oberkochen, Germany) at 120 kV. Images were collected using a ProScan CCD camera.

### Copper site data assembly

The raw reads were filtered for duplicates, sequences matching the Nextera XT adapters, and transposase sequences, and were then quality trimmed using CLC (quality limit 0.05, ambiguous limit 3, adapter trimming, minimum read length 50). PhiX reads not removed by the Illumina MiSeq reporter software (version 3) or through duplicate removal were filtered by mapping all reads to PhiX-174 using RAMICS (Wright and Travers, 2014). Similarly, reads matching the human genome (Hg19; http://tinyurl.com/jay436s) were filtered using consecutively Bowtie2 and RAMICS. This filtered sequence data has been submitted to the European Nucleotide Archive (ENA) under accession number PRJEB13486. The assembly workflow is visualized in Figure S2. Reads were assembled using CLC with stringent assembly settings (automatic word size equaling 22, mismatch cost 2, insertion cost 3, deletion cost 3, min contig length 200, with length and similarity fraction for read mapping equaling 0.95%, both), to generate a primary assembly. Our analyses of the simulated datasets had shown that CLC reconstructs excessively covered genomes into multiple copies and subsequences of nearly 100% sequence identity. We identified such incomplete assemblies through network analysis: all sequences of the primary assembly were aligned against each other using MUMmer and sequence names of identical sequence pairs were clustered using network analysis (in-house R-script). With this approach we found one cluster of 286 contigs. Read mapping using RAMICS showed that these contigs combined 3.6 M reads. We also mapped the remaining reads against another 11 contigs that had an average read coverage above 5000 and were represented by single copies. We then separately assembled all unmapped reads (sub-assembly 1) and the reads that had mapped to the contig cluster (sub-assembly 2). The sub-assembly 1 was then filtered once more for human sequences using BLASTn (BLAST+ suite: Camacho et al., 2009) against the human genome. The final assembled dataset was limited to sequences ≥300 nt and contained 20,097 contigs and scaffolds from subassembly 1, one contig from subassembly 2, and 8 of the highly covered contigs from the primary assembly.

### Copper site data annotation

The annotation workflow for the copper site protein dataset is shown in Figure S3. First, all contigs and scaffolds ≥300 nt were submitted to MetaVir (Roux et al., 2011) for gene prediction and taxonomic classification. MetaVir annotates a protein as “viral” if it matches a protein from the viral subset of the RefSeq protein database with a bit-score above 50. All protein sequences predicted by MetaVir were also compared against the non-redundant protein database (NCBI-nr) and the Refseq protein database (RefSeq-P) from NCBI using BLASTp (BLAST+). The BLAST+ algorithms were executed to provide a tabular output with information on the subject title and subject taxonomy with a preliminary *e*-value threshold of 0.1e-3, and the top hit was used for protein annotation. We then compared the three protein annotations (MetaVir, NCBI-nr, RefSeq-P) and selected the highest scoring annotation for “score-based taxonomic kingdom assignment.” The “final taxonomic kingdom assignment” was “Viruses” if (a) the MetaVir annotation had the highest score (in this case, the respective protein annotations were used to construct a “viral keyword” dictionary); (b) the NCBI-nr and/or RefSeq-P annotations had the highest score, taxonomically classified the protein to “Viruses,” and the *e*-value was below 1.0e-04 (min bit-score 45); (c) the NCBI-nr and/or RefSeq-P annotations had the highest score, the subject title contained a “viral keyword,” and the *e*-value was below 1.0e-04 (min bit-score 45); or (d) the Pfam annotation indicated viral taxonomy. The “final taxonomic kingdom assignment” was “putative Viruses” if the NCBI-nr and/or RefSeq-P annotations had the highest score, indicated viral taxonomy based on taxonomic classification or contained a “viral keyword,” and the *e*-value was above 1.0e-04. Alternatively, proteins were classified as “archaea,” “bacteria,” or “eukaryota” (min *e*-value 1.0e-04) based on NCBI-nr and/or RefSeq-P annotations or remained unclassified. The results were extensively verified through manual annotation (Vm flag). To determine the lowest common ancestry (LCA) affiliation for the viral proteins (V/Vm), we repeated BLASTp against the RefSeq viral protein database and analyzed the results using MEGAN5 (Huson et al., 2007) with min score 45, max expected 1.0e-4, top percent 10, min support 1, and LCA percent 100. The taxonomic IDs provided by MEGAN5 were submitted to phyloT (http://phylot.biobyte.de/index.html) and the resulting tree was visualized using iTOL (Letunic and Bork, 2007). In addition, all contigs and scaffolds ≥300 nt were compared against the non-redundant nucleotide (NCBI-nt) and the RefSeq genomes (RefSeq-G) databases from NCBI using BLASTn (BLAST+). Protein annotations of the “final taxonomic kingdom assignments” were collated by contig and then compared with the corresponding BLASTn annotations to obtain indications on contig taxonomy. For genome comparisons, conducted with *Thalassotalea loyana* phage BA3 and contig_13, we used Easyfig v2.1 (Sullivan et al., 2011).

### Screening copper site data for cellular contamination

The SILVA_128_LSUParc and the SILVA_128_SSUParc fasta files (rRNA genes for large and small subunits, SILVA version 128) were obtained from http://ftp.arb-silva.de/release_128/Exports. Both, the 13.9 M raw reads, as well as the 6.9 M quality filtered reads were mapped to the SILVA database using BLASTn (BLAST+) using a min bit-score threshold of 80. The extensive dataset of viral and cellular marker proteins (Laffy et al., 2016), was downloaded from XXX, and all proteins predicted on the final copper site assembly were mapped using BLASTp (BLAST+) with a maximum *e*-value threshold of 1.0e-10.

### Phylogenetic analyses

For phylogenetic analyses of putative circovirus-like replication-associated (pRepAs) and capsid (pCAPs) proteins, we used MAFFT (Katoh and Standley, 2013) to align the selected protein sequences from our dataset with all matching sequences from the NCBI-nr and RefSeq-P databases (max *e*-value 10). If those proteins originated from metagenomic studies, we used MAFFT to generate preliminary alignments and neighbor-joining trees with all circovirus-like pRepAs and pCAPs from the respective studies (downloaded from UniProt), retaining those protein sequences that showed informative sequence similarities to our proteins. The final alignments were generated using MAFFT, the final phylogenetic trees were generated using the RAxML BlackBox web server (Stamatakis et al., 2008) with default settings and Maximum Likelihood search for best scoring tree after bootstrapping (100) turned on. Figtree was used for tree visualization (Rambaut, 2008).

### Viral host identification assay

The copper site virome assembly indicated presence of a *T. loyana* BA3-like virus in the environmental sample. To verify this, we conducted PCR with the primers TH5For (5′-AGGCGCTAACCTGTGGTCAC-3′) and TH5Rev (5′-CGTTCATGTGTGGCGCTACA-3′), expected to amplify a 5 kb region from the corresponding contig_13. We prepared 50 μl reactions using 200 ng of template DNA, 0.5 μM of the primers, 1X Phusion buffer, 200 μM dNTPs, and 0.02 U/μl Phusion DNA polymerase from Thermo Scientific; and used the following cycling conditions: 1 cycle of 98°C for 3 min; 34 cycles of 98°C for 10 s, 58°C for 30 s, and 72°C for 1 min; followed by 72°C for 10 min and refrigeration at 4°C.

Potential hosts for the *T. loyana* BA3-like virus were investigated by challenging known species of *Thalassomonas/Thalassotalea* with the environmental virus fraction and by PCR screening. *T. loyana* (ID: 280483), *Thalassomonas viridans* XOM25 (ID: 137584), *Thalassotalea agariperforans* (ID: 864068), *Thalassotalea ganghwensis* (ID: 221989), *Thalassotalea agarivorans* (ID: 349064), *Thalassomonas actiniarum* (ID: 485447), and *Thalassomonas haliotis* (ID: 485448) were tested. Each strain was assayed for virus infection using the standard soft agar overlay technique. Briefly, overnight *Thalassomonas/Thalassotalea* cultures were used to inoculate 50 ml of fresh marine broth (1% v/v) and grown until O.D. 600 reached 0.4. Growth temperatures ranged from 20 to 35°C depending on the strain (Yi et al., 2004; Jean et al., 2006; Thompson et al., 2006; Hosoya et al., 2009). Then, 200 μl of the resulting culture were added to 1.5 ml tubes containing 100 μl of serially diluted environmental TFF virus fraction and incubated at room temperature for 10 min. The mixture was added to 3 ml broth containing 0.5% bacteriological agar and spread evenly on agar plates. The plates were checked for the presence of plaques every 24 h. To detect possibly low levels of viral infection, total DNA from virus challenged bacterial cells was used to amplify a region of the large terminase subunit (50 μl reactions using 200 ng of template DNA, 0.5 μM of the primers TerLF (5′-TGGGAAAACCTAACAGATGCC-3′) and TerLR (5′-ATGCAAGCCCATTTGCTGAAG-3′), 1X Phusion buffer, 200 μM dNTPs, and 0.02 U/μl Phusion DNA polymerase from Thermo Scientific). The following conditions were applied: 1 cycle of 98°C for 3 min; 34 cycles of 98°C for 10 s, 65°C for 30 s, and 72°C for 1 min; followed by 72°C for 10 min and refrigeration at 4°C.

## Results

### Assembly of simulated viromes

We tested five assembly programs for their accuracy in reconstruction of viromes unaffected by sample processing (SIM1), and virome datasets with sample processing biases such as severe genome fragmentation (SIM2) and excessive read coverage for selected small circular virus genomes (SIM3). At constant read numbers, increased read length (300 vs. 80 nt) improved assembly accuracy and genome recovery, but did not change the performance of the assemblers relative to each other. Below we show the results for the 300 nt datasets, the results for the 80 nt datasets are provided in Table S1.

Sim1: This dataset consisted of 4.5 M read pairs from 572 complete viral genomes representing a published simulated virome (Vázquez-Castellanos et al., 2014). The results for the five assemblers are summarized in Table 1. The highest number of recovered genomes (195) was achieved using CLC. This assembler also had the highest N50 (~27 kb). This was associated with top performance in assembly of large scaffolds (99 ≥ 50 kb), 90% of which were reconstructed correctly. Comparable results were obtained with IDBA-UD, which showed exceptional assembly precision for most scaffold length categories and successfully reconstructed 147 genomes. In comparison, SPAdes correctly reconstructed a larger number of scaffolds <50 kb, resulting in the longest total correctly assembled sequence length (31.5 Mb). However, for scaffolds ≥50 kb assembly precision was considerably reduced (16% misassembled sequences). This trend was even more accentuated with RayMeta, which reconstructed only 44 correct scaffolds ≥50 kb. ABySS, which had performed comparatively well with the 80 nt dataset (Table S1), was outperformed by the other assemblers in most measured parameters.

**Table 1:** **Assembly results for the simulated virome dataset SIM1 for all contigs larger than 500 nt**.

	**CLC**	**IDBA-UD**	**RayMeta**	**SPAdes**	**ABySS**
Read pairs mapped concordantly to assembly (%)	97	74	91	97	50
>1 times to same scaffold (%)	0.1	0.0	8.8	0.5	5.5
>1 times to different scaffolds (%)	8.1	4.2	6.1	6.0	1.3
Number of scaffolds (% misassembled)	7545 (3)	9454 (1)	8333 (1)	10461 (1)	8179 (2)
500–999 nt	3400 (1)	4336 (1)	4016 (0)	5288 (1)	3974 (2)
1–1.999 kb	1946 (2)	2655 (1)	2127 (0)	2811 (0)	2190 (2)
2–4.999 kb	1207 (4)	1556 (1)	1285 (0)	1416 (1)	1336 (1)
5–9.999 kb	426 (5)	458 (1)	451 (1)	411 (4)	374 (1)
10–19.999 kb	227 (5)	168 (2)	224 (4)	212 (9)	161 (4)
20–49.999 kb	240 (7)	189 (3)	169 (10)	229 (7)	110 (6)
50–99.999 kb	68 (10)	62 (5)	47 (30)	66 (14)	25 (60)
100–199.999 kb	27 (7)	26 (19)	12 (17)	24 (21)	9 (44)
≥200 kb	4 (25)	4 (0)	2 (50)	4 (25)	0 (na)
Max scaffold length:	277,192	257,672	249,422	262,666	178,000
N50:	27,249	15,340	11,876	17,270	5723
Scaffolds correctly mapped to genomes	7360	9389	8266	10,338	8022
Scaffolds correctly mapped to genomes (%)	98	99	99	99	98
Number of misassembled scaffolds	185	65	67	123	157
Total correctly assembled length (Mb)	30.1	31.0	24.8	31.5	19.0
Genomes hit	558	557	531	554	490
Genomes recovered	195	147	124	181	45
ALE score [0,1]	0.2	0.6	0.3	0.3	1.0

Sim2: With this dataset we tested the different assemblers for their accuracy in reconstructing short distinct fragments. We fragmented the genome sequences of the 572 viruses into 2 kb pieces, discarded every second sequence and generated 4.5 M read pairs from the remaining 11,743 sequence fragments. Allowing for a maximal over-length of 5%, any scaffolds ≥2.1 kb were considered misassembled. The results for this dataset are summarized in Table 2. With this dataset, ABySS performed best in terms of assembly precision: 90% of the scaffolds were equal to or smaller than the expected maximum length of 2 kb, and only 10% of those were misassembled. The other assemblers produced high numbers of scaffolds ≥2.1 kb and misassembly rates for the investigated smaller scaffold fractions were generally high (16–43%). Of those four assemblers, CLC performed best in terms of precision (lowest misassembly rates for all length categories), while IDBA-UD prevailed in terms of numbers, resulting in the highest number of correctly reconstructed scaffolds (9136) and the longest total correctly assembled sequence length (13.3 Mb).

**Table 2:** **Assembly results for the simulated virome dataset SIM2 for all contigs larger than 500 nt**.

	**CLC**	**IDBA-UD**	**RayMeta**	**SPAdes**	**ABySS**
Read pairs mapped concordantly to assembly (%)	91	62	67	95	43
>1 times to same scaffold (%)	0.1	0.0	0.3	42.0	0.8
>1 times to different scaffolds (%)	8.3	3.8	6.6	4.8	0.8
Number of scaffolds (% misassembled)	11,991 (38)	19,346 (46)	14,196 (51)	12,414 (47)	11,279 (18)
500–999 nt	4268 (20)	5593 (30)	4389 (21)	5826 (25)	5093 (9)
1000–1499 nt	1684 (30)	2513 (43)	1881 (37)	2040 (37)	1914 (14)
1500–2099 nt	3373 (17)	5955 (16)	3050 (22)	1475 (42)	3217 (8)
≥2100 nt:	2666 (100)	5285 (100)	4876 (100)	3073 (100)	1055 (100)
Max scaffold length:	226,834	47,930	24,446	365,072	134,592
N50:	4023	2001	2454	14,846	2001
Scaffolds correctly mapped to genomes	4697	9136	5254	4524	5586
Scaffolds correctly mapped to genomes (%)	39	47	37	36	50
Number of misassembled scaffolds	7294	10,210	8942	7890	5693
correctly assembled length (Mb)	6.3	13.3	6.9	4.6	6.9
Genomes hit	4592	8267	4995	4030	5120
Genomes recovered	1809	4420	1881	451	1517
ALE score [0,1]	0.4	0.8	0.7	0.3	1.0

Sim3: Preliminary analyses of the copper virome sequencing data indicated severe excessive read coverage for selected circovirus-like genomes in the dataset. Therefore, Sim3 consisted of 4.5 M read pairs generated from the above genomes with previous relative read coverage and five additional circovirus genomes with exceptionally high relative read coverage. The results are summarized in Table 3. CLC again produced the highest number of correctly assembled large scaffolds (only 1 of the 47 scaffolds ≥50 kb was misassembled). It also recovered the highest number of genomes (148), including the five highly covered circoviruses. We observed that these highly covered circovirus genomes had been reconstructed by CLC into multiple copies and subsequences of nearly 100% sequence identity. The next best assembler in terms of precision was IDBA-UD, which outperformed CLC in correct assembly of smaller scaffolds (misassembly rate for scaffolds <50 kb only 0.7%). It correctly reconstructed 40 scaffolds ≥50 kb (10% misassembly rate), and recovered 111 genomes. Noticeably, with this dataset IDBA-UD used only 31% of the reads, and did not reassemble the highly covered circoviral sequences. As with SIM1, SPAdes, and RayMeta showed exceptional average assembly precision (average misassembly rates 0.9 and 0.6%, respectively), which, however, masked high misassembly rates for the few large scaffolds ≥50 kb (15 and 21%, respectively). Although SPAdes produced the longest correct total sequence length (~26 Mb), this was mainly due to the correct assembly of high numbers of smaller scaffolds. Besides CLC, SPAdes was the only other assembler that recovered the circoviral genomes. ABySS was again outperformed by all other assemblers for most measured parameters.

**Table 3:** **Assembly results for the simulated virome dataset SIM3 for all contigs larger than 500 nt**.

	**CLC**	**IDBA-UD**	**RayMeta**	**SPAdes**	**ABySS**
Read pairs mapped concordantly to assembly (%)	89	31	40	92	18
>1 times to same scaffold (%)	0.2	0.0	29.1	1.1	1.1
>1 times to different scaffolds (%)	2.8	3.1	3.5	1.4	0.9
Number of scaffolds (% misassembled)	9068 (2)	9543 (1)	8430 (1)	12,231 (1)	7248 (1)
500–999 nt	4433 (1)	4537 (1)	4571 (0)	6657 (0)	4067 (1)
1–1.999 kb	2449 (2)	2694 (1)	2295 (0)	3280 (1)	1873 (1)
2–4.999 kb	1352 (3)	1548 (1)	1041 (1)	1573 (1)	867 (1)
5–9.999 kb	456 (3)	436 (1)	266 (2)	374 (4)	226 (1)
10–19.999 kb	166 (5)	154 (2)	117 (4)	152 (6)	130 (4)
20–49.999 kb	165 (5)	132 (6)	111 (8)	155 (7)	75 (8)
50–99.999 kb	35 (3)	29 (10)	23 (22)	28 (14)	7 (43)
100–199.999 kb	11 (0)	12 (8)	6 (17)	11 (18)	3 (67)
≥200 kb	1 (0)	1 (0)	0 (na)	1 (0)	0 (na)
Max scaffold length:	244,834	244,953	163,001	245,076	141,829
N50:	8073	5958	5262	4759	3766
Scaffolds correctly mapped to genomes	8900	9471	8380	12,126	7158
Scaffolds correctly mapped to genomes (%)	98	99	99	99	99
Number of misassembled scaffolds	168	72	50	105	90
Total correctly assembled length (Mb)	25.0	24.2	18.1	26.1	13.6
Genomes hit	565	552	514	559	479
Genomes recovered	148	111	83	126	23
ALE score [0,1]	0.2	0.9	0.8	0.2	1.0

### Copper site data assembly

The copper dataset consisted of 13.8 M raw sequencing reads (6.9 M read pairs). Despite high quality, a large number of reads was lost during preassembly filtering: 4.3 M reads were duplicates, 1.3 M reads matched PhiX-174, and 1.1 M reads matched the human genome. The remaining 6.9 M high quality reads were used for assembly with CLC, which performed best in recovery of viral genomes. The results are summarized in Table 4. The primary assembly of the reads produced 38,365 contigs longer than 200 nt, and the largest contig was 35.5 kb. As discovered in our SIM3 analyses, CLC sometimes reconstructs highly covered sequences into several copies and subsequences. We therefore aligned the assembly to itself and investigated it for replicated sequences using network analyses. This lead to the identification of a contig cluster of 286 nearly identical sequences, assembled from a total of 3.6 M reads. Another 11 contigs that had an average read coverage >5000 were present as single copies. In total, these sequences had combined 67% (4.6 M reads) of the dataset. After removing these reads, we proceeded with the separate assembly of the 2.3 M unmapped reads (sub-assembly 1), and the 3.6 M reads that had mapped to the contig cluster (sub-assembly 2). Despite the significantly lower read number, sub-assembly 1 produced a similar number of contigs, a comparable contig length distribution, and a marginally higher N50 than the primary assembly. Sub-assembly 2 produced 135 contigs, which really represented only two different sequences: a potentially circular DNA sequence of 1308 nt and a linear contig consisting of repeats. The remaining 133 sequences were shorter versions of these two contigs.

**Table 4:** **Assembly results for the Namib Desert copper-site dataset for all contigs larger than 200 nt**.

	**Reads used**	**Contigs**	**Assembled reads**	**% reads assembled**	**N50**	**Max**
Primary assembly	6,877,654	38,365	5,356,821	77.9	913	35,461
Sub-assembly 1	2,241,214	35,966	1,562,711	69.7	961	35,670
Sub-assembly 2	3,629,636	135	3,385,364	93.3	–	1835

### Copper site data annotation

A total of 20,106 scaffolds ≥300 nt were submitted to MetaVir for gene prediction and annotation. The program predicted 33,254 ORFs, and assigned 4027 ORFs from 3382 scaffolds to viral taxonomic groups based on BLAST matches to the viral subset of the RefSeq database. Our subsequent protein annotations using the NCBI-nr and RefSeq-P databases and manual verifications could only confirm viral origin for 1274 proteins. Of these, 577 had Pfam domain annotations, many of which indicated viral origin (e.g., terminase, phage-integrase, phage portal, phage capsid). The remaining 2753 proteins (68%) had better matches to archaeal, bacterial, or eukaryotic proteins in the NCBI-nr and/or RefSeq-P databases, and Pfam annotations were not virus-related. Only 305 of these proteins were found on contigs that encoded at least one confirmed viral protein, i.e., may potentially represent viral genes. For 51% of these proteins the score from MetaVir was less than half of the top score. To avoid false viral annotation, we did not include these proteins into our final viral protein dataset. Furthermore, we identified eight proteins that had Pfam annotations indicating viral origin, and 392 proteins that had matched viral proteins in the RefSeq-P and/or NCBI-nr databases (max 1.0E-4, min bit-score 45), but had not been detected by MetaVir. Our final dataset of predicted viral proteins therefore consisted of 1674 sequences (Table S2).

**Figure 1:**
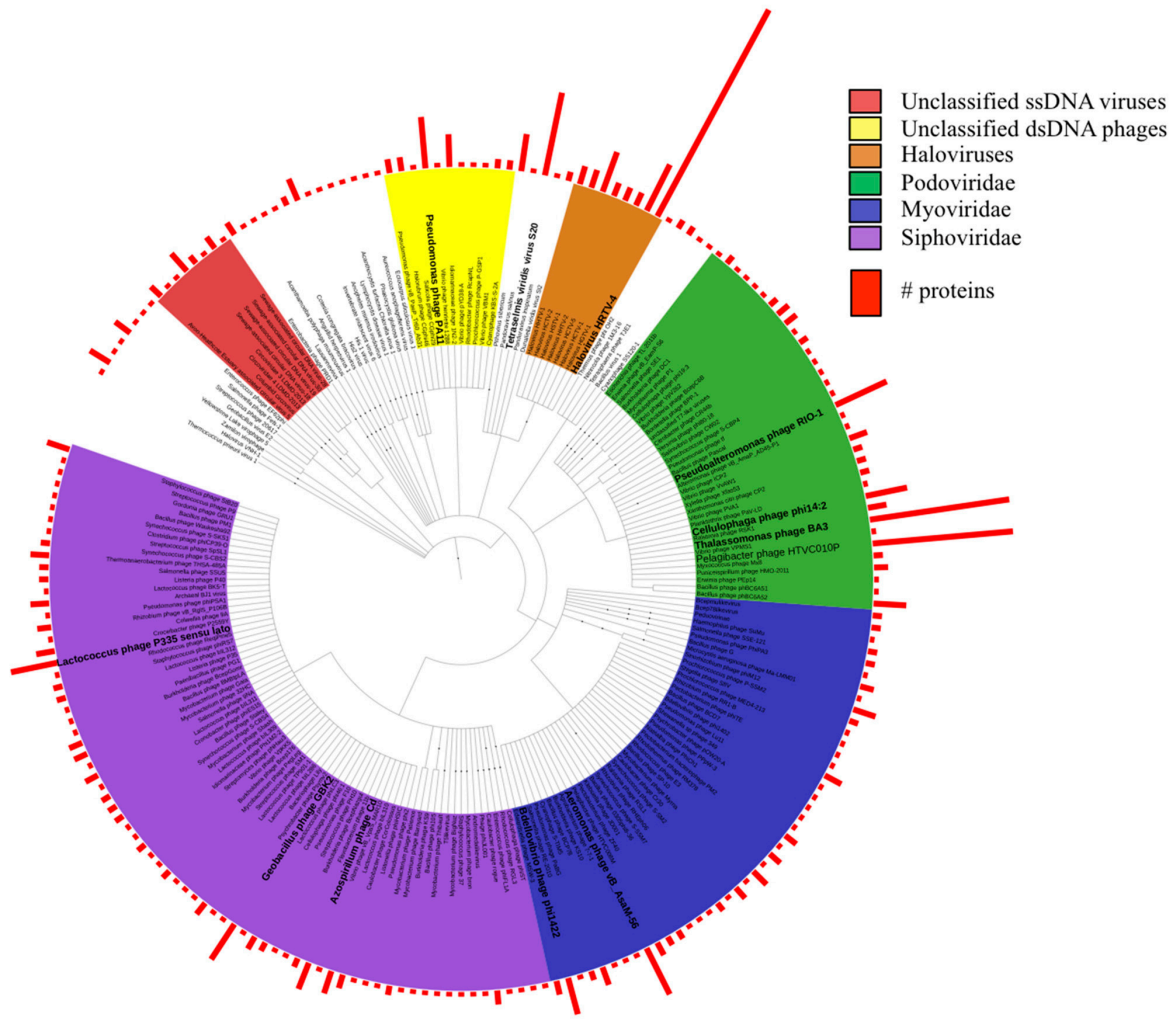
**Taxonomic tree of all viral protein sequences discovered in this study**. Proteins were assigned taxonomic IDs using the lowest common ancestor method. Bars depict numbers of proteins assigned to the corresponding taxa.

To determine the LCA affiliation for these proteins, we repeated BLASTp against the viral protein RefSeq database and analyzed the results using MEGAN5. Taxonomic affiliations were obtained for 1288 proteins and the results indicated a diverse spectrum of viral species in the dataset (Figure 1). The majority of the proteins (748) were assigned to Caudovirales, including siphoviridae (259), podiviridae (213), and myroviridae (159) species, supporting our TEM results (Figure S4). Most species were represented by a single protein. However, within the unclassified podoviridae, 30 proteins were affiliated with *Pelagibacter* phage HTVC010P, another 30 proteins with *Thalassomonas* phage BA3, 12 proteins with *Pseudoalteromonas* phage RIO-1 and nine proteins with *Cellulophaga* phage phi14:2. Among the siphoviridae, higher protein numbers were found for *Azospirillium* phage Cd (6), *Geobacillus* phage GBK2 (9), and for diverse *Lactococcus* phages. Of the myoviridae species *Aeromonas* phage vB_AsaM-56 had the highest protein count (11). Among the unclassified dsDNA viruses, archaeal viruses prevailed by far: 197 proteins were assigned to haloviruses, with *Halovirus* HRTV-4 representing the most common top hit. Two thirds of the quality filtered reads from the copper site dataset (4.1 M) assembled into just 35 contigs that encoded proteins with sequence similarity to proteins from small circular viruses. This included the 1308 nt potentially circular contig from sub-assembly 2, which combined 3.6 M reads. It encoded one geminivirus-like capsid protein (Pfam: PF00844) and two other ORFs that could not be annotated. Since the replication-associated protein could not be identified, it remains unclear whether this particular contig represents a complete viral genome. Most of the other circovirus-like contigs encoded proteins with similarity to sequences from recent virome studies on seawater samples from Tampa Bay, Florida, USA (McDaniel et al., 2014), mollusks from the Avon Heathcote estuary in New Zealand (Dayaram et al., 2015), and sewage-associated circular viruses (Kraberger et al., 2015).

### Phylogenetic analyses on circovirus-like proteins

**Figure 2:**
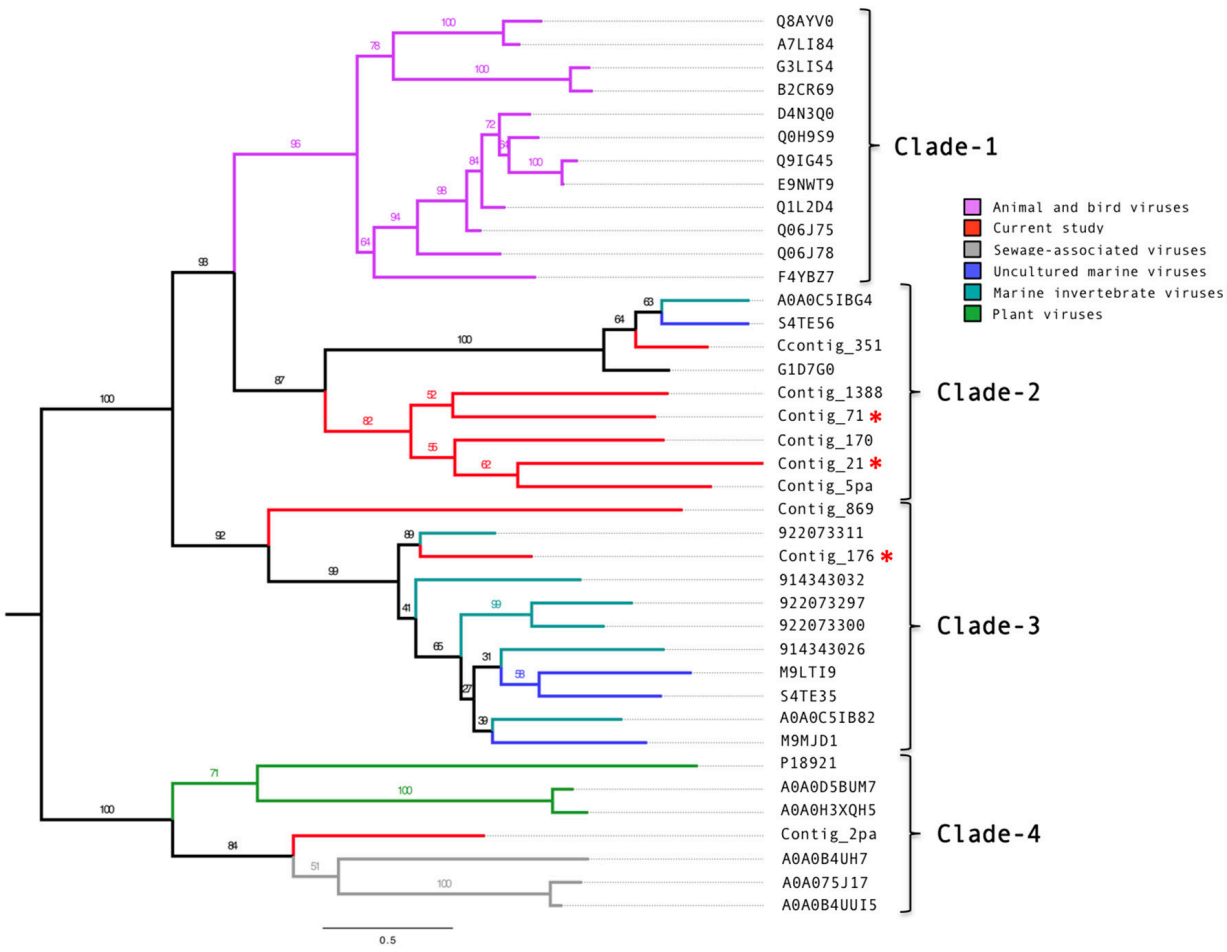
**Phylogenetic tree of the circovirus-like replication-associated proteins**. Alignments were generated using MAFFT, the phylogenetic tree was produced using the RAxML BlackBox, the tree was visualized using FigTree. The asterisk serves to identify the replication and capsid proteins identified in this study.

Manual annotation of the contigs with circoviral-like proteins allowed identification of nine putative replication-associated proteins (pRepAs). All but the pRepA from contig_176 contained the viral replication (PF02407) and the RNA-helicase (PF00910) domains (Figure S5). Our phylogenetic analyses (Figure 2) showed that these proteins differed from described animal and bird circoviral RepAs (clade-1), forming three distinct, well-supported clades. Most of the pRepAs discovered in this study formed a monophyletic sub-clade within clade-2, co-segregating with a sub-clade containing the pRepA from contig_351 as well as one pRepA from a marine water study (Labonté(Labonté and Suttle, 2013)), a mollusk (Dayaram et al., 2015) and a bat feces virome study (Ge et al., 2011), respectively. In all proteins of clade-2, the Rep motif I was unrecognizable, while the motifs II (H.Q) and III (Y.KD/E) were conserved, resembling those described for animal and bird circoviral RepAs. Clade-3 contained the pRepA from contig_176, six RepAs from diverse marine invertebrates and three RepAs from uncultured marine viruses. In these sequences, all three RepA motifs (I: FTINN, II: HLQG, III: YCKKD) were conserved, again resembling those described for animal circoviral RepAs. For the pRepA of contig_2pa, the Rep motifs I (FLTF), II (HLH), and III (YCMKD) resembled those of plant geminiviruses. However, it did not contain the recently described geminivirus replication (GRS) domain. Accordingly, phylogenetic analyses placed this protein, as well as the three pRepAs from the Sewage-associated circular DNA viruses 18, 14, and 28 (Kraberger et al., 2015) into clade-4 which harbored three plant viral RepAs (chloris striate mosaic virus, mulberry mosaic dwarf associated virus and Mulberry crinkle-associated virus).

**Figure 3:**
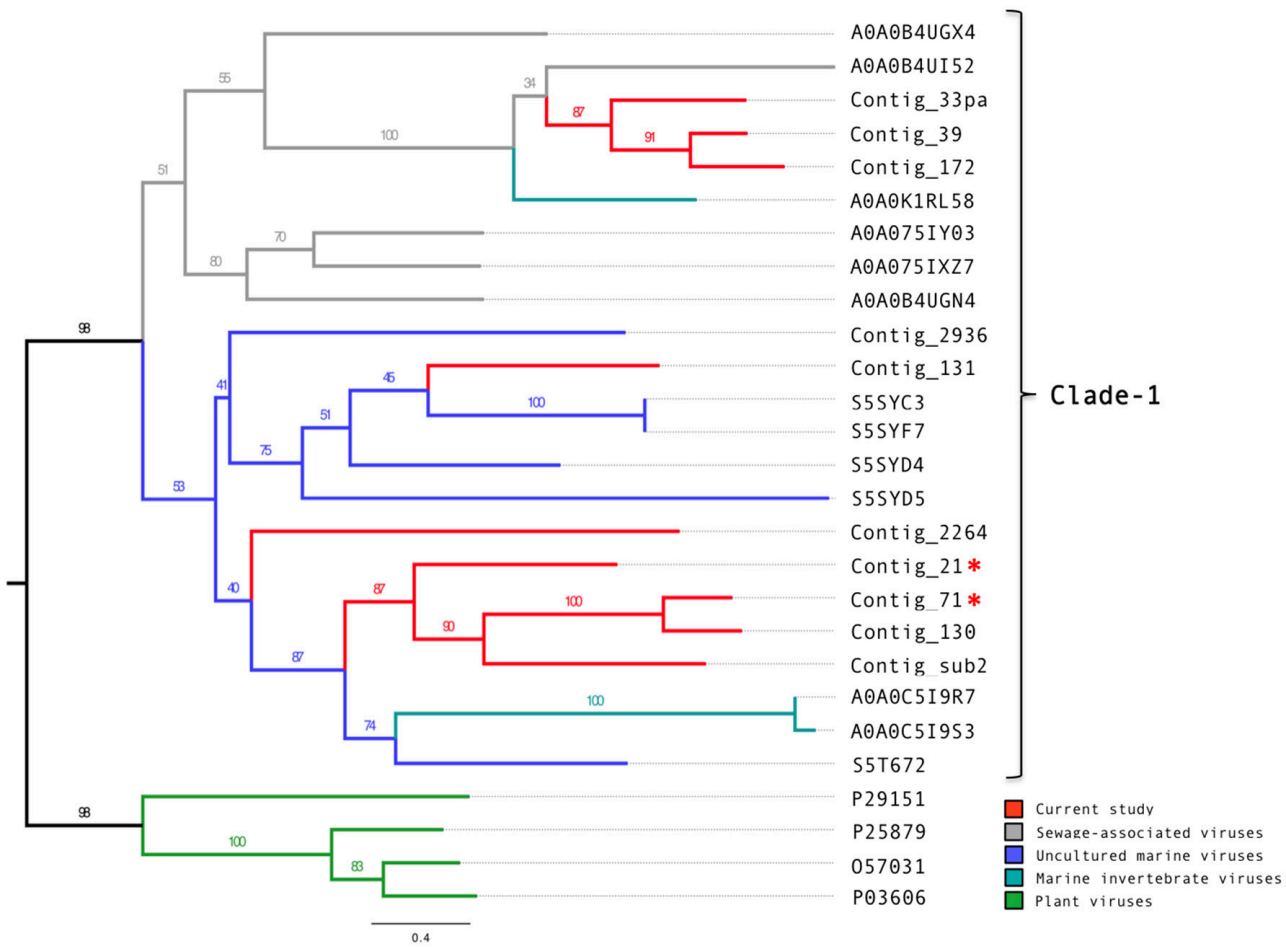
**Phylogenetic tree of the circovirus-like capsid proteins**. Alignments were generated using MAFFT, the phylogenetic tree was produced using the RAxML BlackBox, the tree was visualized using FigTree. The asterisk serves to identify the Replication and capsid proteins identified in this study.

Of the 14 putative putative circoviral-like capsids (pCAPs) discovered in this study, nine shared sufficient sequence similarity to each other and to known circoviral-like putative capsid proteins to generate a phylogenetic tree (Figure 3, Figure S6). Clade-1 contained the pCAPs from this study, five pCAPS from a sewage virome study (Kraberger et al., 2015), six pCAPs from a seawater virome study (Tampa Bay, FL, USA; McDaniel et al., 2014), two pCAPs from the Avon Heathcote Estuary mollusk virome study (Dayaram et al., 2015) and one putative capsid from a fiddler crab virus. Within clade-1, the sewage virus pCAPs and the seawater virus pCAPs formed separate sub-clades, but branch support was low. Clade-2 contained selected plant satellite viruses (Maize white line mosaic Satellite virus and three Satellite tobacco necrosis viruses). None of the investigated sequences showed similarity to any of the known animal or bird circoviral capsid proteins.

### Novel small circovirus-like genomes

Here, we describe three novel circovirus-like genomes identified in this study (Table 5). The pRepAs and the pCAPs of these genomes are included in the above analyses and show that contig_21 and contig_71 are potentially closely related viral species as both the pRepAs and the pCAPs co-segregate on the respective phylogenetic trees. Contig_176 represents a more distantly related viral species, with a pRepA that shows similarity to invertebrate circoviral pRepAs.

**Table 5:** **Annotation table for novel circoviral-like genomes discovered in this study**.

	**Annotation**	**Coordinates**			
Contig_21	ORF1	128–937 (−1)	PF00844	44% id to hypothetical protein from Circoviridae 3 LDMD-2013	putative gemini coat protein
	ORF2	1127–2130 (+2)	PF02407, PF00910	38% id to replication-associated protein of the Avon-Heathcote Estuary associated circular virus 5	putative viral replication protein
	ORI	1078–1115	GACC**AGTATTAC** flanked by a perfect 13 nt palindromic repeat		
Contig_71	ORF1	109–1410 (+1)	PF02407, PF00910	40% id to the replication-associated protein of the Avon-Heathcote Estuary associated circular virus 5	putative viral replication protein
	ORF2	1572–2360 (−3)	PF00844	35% id to a hypothetical protein from Circoviridae 3 LDMD-2013	putative gemini coat protein
	ORI	42–83	CT**AGTATTAT** flanked by an imperfect 15 nt palindromic repeat		
Contig_176	ORF1	50–955 (+2)	PF02407	59% id to the putative replication initiation protein of the *Gammarus* sp. amphipod associated circular virus	putative viral replication protein
	ORF2	1187–1951 (−3)	–	–	–
	ORI	1–33	CCT**ATTATTAC** flanked by a perfect 11 nt palindromic repeat		

*The nonanucleotide sequence where ssDNA synthesis is initiated is shown in bold*.

### Novel *Thalassotalea loyana* phage Ba3-like viral genome

The largest contig of the assembly (Contig_13) represented a potentially complete 35.461 nt linear viral genome with a uniform high average read coverage [1221]. It encoded 47 ORFs, with ORFs 1–39 located on one, and ORFs 40–47 on the complementary DNA strand (Table S3). Gene annotations using BLAST and Pfam predicted two putative early genes (ORF42, ORF40), four putative late genes (ORF2, ORF8, ORF17, ORF18), one structural gene (ORF12), and one gene that may potentially be involved in host lysis (ORF21). A 5 kb region of the BA3-like virus could be amplified from the environmental fraction confirming presence of the virus in the sample. Furthermore, end sequence analysis of the amplified product showed 100% sequence identity to Contig_13, providing confidence in the assembly. However, host infection assays with seven *Thalassomonas/Thalassotalea* species using the environmental virus fraction failed to produce plaques, and PCR analyses of virus challenged bacterial cells did not amplify the target region.

**Figure 4:**
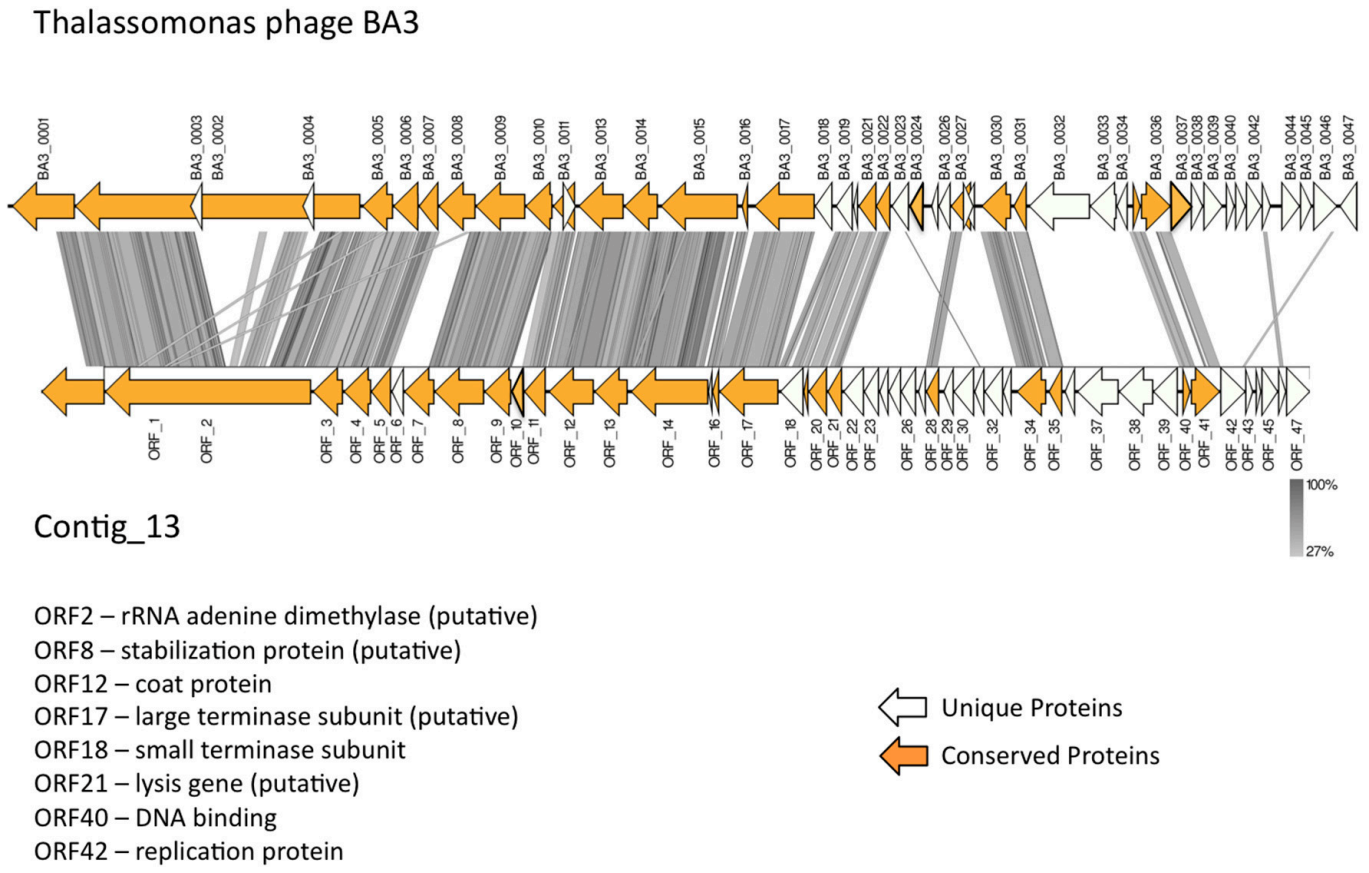
**Comparison of the published *Thalassotalea loyana* phage BA3 genome and contig_13, representing a potentially complete BA3 phage–like genome**.

In total, 22 ORFs had top matches to *T. loyana* phage BA3, including the ORF with the highest score (used for taxonomic assignment by MetaVir). Sequence comparisons between Contig_13 and BA3 indicated high levels of conservation for gene order, gene orientation, and protein sequences (average percent identity between the 22 ORFs was 64%) for the corresponding 5′ regions of the two sequences (Figure 4). However, most of the early genes of BA3 are located in the 3′ region of the genome and had no homologs on contig_13. Further investigations are necessary to identify the host of the Contig_13 virus.

### Non-viral sequences in the copper site dataset

Contig annotation indicated that at least 5.1 M reads of the quality filtered dataset originated from viral DNA. However, we also found 1.1 M human reads, 50% of which assembled into contigs of up to 8 kb. Another 0.6 M quality filtered reads assembled into sequences that were annotated as archaeal, bacterial or eukaryotic. This degree of contamination was not evident when screening the reads against the complete SILVA database: 0.06% of the reads from the raw dataset and 0.1% of the reads from the filtered dataset mapped to rRNA genes at min bit-score 80, indicating negligible amounts of cellular contamination as defined by Roux et al. (2013). Furthermore, at max 1.0E-10, only 577 of the 33296 predicted proteins (1.6%) mapped to cellular protein sequences from the extensive marker gene database published by Laffy et al. (2016).

We used this ancillary environmental data to identify new prophage genes. We had obtained substantial genome coverage for a *Limnobacter* strain: 1429 contigs (cumulative length 2 Mb) matched the *Limnobacter sp*. MED105 genome at 85% identity over 95% of their length; and 3694 proteins had top hits to this bacterium. Twelve proteins had BLAST and Pfam annotations indicating viral origin (e.g., toprim proteins, phage integrase).

## Discussion

Initial analyses of the copper dataset indicated fragmented representation of the original genomes and excessive read coverage for a number of small viral genomes. Previous studies with simulated datasets had not addressed these particular data issues. To identify a suitable assembly program, we simulated datasets that mimicked the copper-site data challenges, and compared performance of five assemblers that had previously shown best results in metagenome reconstruction (de Cárcer et al., 2014; Vázquez-Castellanos et al., 2014; García-López et al., 2015; Laffy et al., 2016). Of particular interest was the assembly precision for different size sequences, which are expected in metagenomes. In the above citations, this is assessed using averaged values for the whole dataset (average contig numbers, N50, %accuracy, %chimeric sequences). However, large scaffolds that are particularly interesting for downstream analyses usually represent a very small fraction of an assembly, and the degree of their misassembly is not discernable based on those values. We therefore increased the resolution of assembly accuracy assessment by analyzing misassembly rates for distinct scaffold size fractions. We also investigated the ability of each assembler to recover genomes as single scaffolds, since multi-scaffold representation of genomes necessitates additional assembly steps that may generate chimeras. Based on these parameters, the CLC Genomics Workbench emerged as the most suitable assembler: it consistently recovered the highest number of genomes, accurately reconstructed large scaffolds, and outperformed most other assemblers in recognition of fragmented genome data (SIM2 virome). Shorter sequences were often misassembled by CLC with the 80 nt datasets, but these could be identified by long stretches of Ns, and filtering sequences with N% ≥ 20% alleviated the problem. With the 300 nt datasets, CLC assembly accuracies for shorter sequences were comparable to those achieved by the other assemblers. IDBA-UD generally excelled in assembly precision, but failed to assemble the 80 nt SIM2 dataset. SPAdes, RayMeta and ABySS had high average assembly accuracy, but often misassembled sequences ≥20 kb (80 nt) and ≥50 kb (300 nt). Only CLC and SPAdes recovered the circoviral genomes with excessive read coverage (SIM3 virome), which were entirely missing in the assemblies generated by IDBA-UD, RayMeta and ABySS. Interestingly, with the SIM3 dataset, these three assemblers used only a fraction of the reads for sequence reconstruction, which may indicate stringent settings for high-frequency kmers in these programs. Since it is likely that virome composition (relatedness of species, variation in genome sizes, genome coverages) affect assembler performance, kmer assessment of environmental datasets may prove a useful tool for choosing a suitable program. With SIM3 we observed that CLC reassembled the highly covered circoviral genomes into multiple copies and subsequences of nearly 100% sequence identity. To minimize such artificial duplicates in the final copper site assembly, we investigated the primary assembly for duplicates using network analysis. We identified one cluster of 286 contigs. Separate reassembly of the corresponding reads produced 135 contigs, which represented only two distinct sequences based on manual verification. One may argue that such duplicates may represent different viral strains, and in fact, the approach could be applied to identify viral strain variants. With the copper site dataset, however, it would be difficult to distinguish computational and laboratorial artifacts from true viral strain variants. Filtering for reads from excessively covered sequences did not improve assembly of the remaining reads, which contrasts with previous reports.

A number of papers document contamination of currently published virome datasets with non-viral sequences. In 2011, Schmieder and Edwards analyzed 202 published microbial and viral metagenomes and found indications for human DNA contamination in 145 datasets. Roux et al. (2013) reported on substantial cellular DNA contaminations found in a quarter of 67 published viromes. According to the authors, all investigated viromes derived from complex matrices (e.g., feaces, gut, coral, insect, and animal samples) contained DNA encoding ribosomal genes, suggesting that purification of viral particles from such environments may be challenging. Soil samples definitely fall into this category. Enzyme-inhibiting compounds not only affect library construction (Carvalhais et al., 2012; Tveit et al., 2014); they can also protect extracellular DNA (eDNA) from degradation, as demonstrated for humic acids (Crecchio and Stotzky, 1998), organic matter (Nguyen et al., 2010), clay (Mao et al., 2014), and extracellular polymeric substances produced in microbial biofilms (Grande et al., 2011, 2014; Rosario et al., 2015). With soil samples, additional purification steps and WGA are sometimes unavoidable to overcome inhibitors and avoid failure of sequencing reactions. Yet, these procedures can introduce biases such as selective purification of DNA from specific organisms, DNA fractionation and selective sequence amplification (Kim and Bae, 2011; Yuan et al., 2012; van Dijk et al., 2014) that will affect representation of the viral and non-viral DNA. Lower target/contaminant DNA ratios result in higher numbers of reads from contaminants in the read dataset, as demonstrated by Lusk (2014), who found that frequencies of contaminating human reads in an *E. coli* study increased with decreasing sample DNA concentrations. In this study, we followed all standard laboratorial practices for virome analyses, including sterile handling of material, microscopic examination of the sample for bacterial cells, TEM to verify presence of viral particles, as well as DNAse and RNAse treatment of the viral particles prior to protein coat digestion (verified through 16S rRNA PCR with positive and negative controls). We therefore hypothesize that trace amounts of human and environmental DNA escaped DNAse treatment and were preferentially amplified by Phi29, leading to the observed contaminations. Phi29 amplification is also the likely reason for the high read duplication rates and the very selective amplification of circovirus-like genomes: previous analyses have shown that this enzyme predominantly amplifies small circular DNA (Kim and Bae, 2011). Therefore, dedicated optimization studies on sample preparation and sequencing methodologies for soil virome analysis (as conducted for human skin and gut viromes by Hannigan et al., 2015 and Džunková et al., 2015, respectively) would help to address these problems.

As follows from above, virome data must be inspected for contaminating DNA, in particular when the samples were obtained from complex environmental matrices. This does not appear to be common practice: 19 of 25 investigated virome studies published since 2014 appear to rely solely on 16S rDNA PCR to detect bacterial contamination and do not mention biocomputational inspection of their data prior to submitting it to MetaVir for annotation. The MetaVir server does not filter for non-viral sequences, but annotates the submitted data using the viral subset of the RefSeq database. In case of contamination, all non-viral proteins showing significant similarity to proteins in the database will be misannotated as viral. This may not only lead to vast overestimation of bacterial genes in viral sequences identified through virome studies (as found for antibiotic resistance genes by Enault et al., 2017), but also result in error propagation in databases. With our dataset, screening reads against rRNA genes and proteins against the most extensive cellular marker gene database published to date (Laffy et al., 2016) did not indicate substantial contamination of the dataset with non-viral DNA. Yet, large scaffolds of non-viral DNA (up to 18 kb) with high nucleotide identity (≥95%) over their entire length (≥95% coverage) were recovered. Two scenarios can explain this result: (a) genomic regions encoding marker genes were absent, i.e., contamination originated from fragments of free DNA that had escaped DNAse treatment and were selectively amplified; or (b) minute amounts of cellular DNA was present, but only genomic regions not encoding marker genes were selectively amplified. The studies by Schmieder and Edwards (2011), Roux et al. (2013), and Lusk (2014) suggest that such type of contaminations may be quiet common in metagenome datasets. These findings indicate that the virome community would benefit from benchmarking studies using simulated and environmental datasets to establish optimal screening and filtering procedures. In fact, when annotated correctly, data from non-viral DNA can provide interesting ancillary information about the sampled environment, as found in this study. Considering the presence of non-viral DNA in the copper site data, we chose a stringent approach to annotate sequences as viral and used the complete NCBI-nr and RefSeq-P databases rather than the viral RefSeq database for annotation (Hurwitz et al., 2016). While this method results in underestimation of viral sequences, it provides higher confidence in the assignment of viral annotations.

Our sequence data from the Namib Desert copper-site indicated a diverse range of dsDNA viruses from all three families of the Caudovirales. Viruses from ubiquitous bacterial species included *Geobacillus, Bacillus*, and *Aeromonas* phages, confirming previous findings for the Namib Desert (Adriaenssens et al., 2015). A number of the detected viruses may be associated with plants and their rhizobial symbionts, since several proteins showed sequence similarity to diverse *Lactococcus* phages and *Azospirillium* phage Cd. A high number of proteins was affiliated with Haloviruses that infect archaea, which is also in agreement with previous findings for this environment (Adriaenssens et al., 2016). Furthermore, 35 contigs encoded proteins that showed sequence similarity to selected circovirus-like and geminivirus proteins. Three novel circovirus-like genomes could be identified. Only few published proteins shared sequence similarity with the putative replication-associated (pRepAs) and the putative capsid (pCAPs) proteins from these contigs. This is not surprising, considering that these small viruses evolve exceptionally fast (reviewed in Krupovic, 2013). Interestingly, most of these published proteins originated from studies of aqueous environments. So, all analyzed pCAPs from this study co-segregated with viral proteins from sewage (Kraberger et al., 2015), marine water (McDaniel et al., 2014), and estuarian/marine invertebrate virome studies (Dayaram et al., 2015; Rosario et al., 2015). These proteins appeared to be distantly related to selected plant viruses and shared no sequence similarity with known circoviral pCAPs from animals or birds. The pRepAs from Contig_176 and Contig_869 co-segregated with pRepAs of circular ssDNA viruses from diverse marine invertebrates and several uncultured marine viruses. The pRepAs from another six contigs formed a well-supported clade with just three published proteins from one marine (Labonté and Suttle, 2013), one mollusk (Dayaram et al., 2015) and one bat feces (Ge et al., 2011) virome study, respectively. Interestingly, the bat sequence YN-BtCV-1 (phylogenetically unplaced in the above study) originates from a roosting site in Yunnan populated by diverse *Myotis* species. Several members (including *M. pilosus*, which is found in this area) are known to eat fish (Stadelmann et al., 2004). It is possible, that the sequences from the mollusk virome and the uncultured marine virus also represent fish circovirus-like species, which would explain the highly-supported proximity of clade-2 to clade-1 (animal/bird circoviral pRepAs) relative to clade-3 (putative invertebrate circoviral pRepAs) and clade-4 (putative geminivirus-like RepAs). However, considering that our circovirus annotations could be biased due to the limited amount of sequence information on circoviral genomes from other habitats, these interpretations remain to be validated.

Yet, other results of this study also link the investigated virome to the marine environment. Contig_13, which represents a potentially complete 35.5 kb viral genome, shares high sequence similarity with *T. loyana* phage BA3, which infects the coral pathogen *T. loyana* (Efrony et al., 2009). Although we could not confirm that *Thalassomonas/Thalassotalea* species are hosts for the identified virus, the extensive degree of conservation between the two genomes may indicate common ancestry. Another 51 protein sequences were taxonomically affiliated with Pelagibacter phage HTVC010P, Cellulophaga phage phi14:2, and Pseudoalteromonas phage RIO-1, all of which infect marine bacteria. Incidentally, these four species all belong to the podoviridae family, which represents a major fraction in marine viromes, but appears to be less common in viromes from non-marine environments including the Namib Desert (Prestel et al., 2008; Adriaenssens et al., 2015). Moreover, a large number of contigs (totaling 2.6 Mb) mapped to the genome of *Limnobacter sp*. MED105, a common marine bacterium. Last but not least, a previous study on viromes from Namib Desert salt pans mentions several contigs that clustered with viral sequences of aquatic origin (Adriaenssens et al., 2016).

Considering that samples analyzed in this study were derived from a soil sample in the Namib Desert, these results are, at first glance, surprising. However, this desert is characterized by seasonal fog from the Atlantic Ocean that reportedly reaches as far as 100 km inland (http://tinyurl.com/zwuky7v). The sample was collected within this distance (~30 km to the south-east of the Gobabeb research station), where “high-fog” is prevalent (Eckardt et al., 2013a). Sampling was conducted at the end of April 2013, just after the typical “high-fog” months in this location (September-March). It is therefore possible that microorganisms and viral particles carried by fog and wind from the Atlantic Ocean represent a sampling fraction of this study. Seasonal variations of this phenomenon could also explain why few viral particles were found on following sampling occasions (April 2014 and 2015, respectively). Investigations on the marine virome diversity in proximate coastal regions may help to verify our hypothesis.

## Author contributions

Lv and MT designed and conceptualized the project and collected the samples, Lv and BK conducted DNA isolation and NGS, UH conceptualized and conducted the biocomputational data analyses and wrote the manuscript, Pv assisted in biocomputational data analyses, IO conducted the Thalassomonas infection studies. All authors read and approved the manuscript.

## Funding

The project was supported by the NRF South Africa through the DST/NRF SARChI programme (UID87326).

### Conflict of interest statement

The authors declare that the research was conducted in the absence of any commercial or financial relationships that could be construed as a potential conflict of interest.

## Supplementary material

Table S1 **Assembly results for the simulated virome datasets SIM1, SIM2, and SIM3 for all contigs larger than 200 nt**.

Table S2 **Annotation table for all proteins discovered in this study comparing MetaVir annotations with those derived from the nr and RefSeq databases from NCBI, considering Pfam**.

Table S3 **Annotation table for the Thalassomonas phage-like genome described in this study (contig_13)**.

Figure S1 **Satellite image of the sampling site (Cu-site) showing its position in relation to nearby copper mines (Hope and Gorob)**. Inset depicts an enlargement of the site showing the trench dug alongside the road with heaps of sampled copper laden material, as well as images of the sampled material (including material with a green copper patina).

Figure S2 **Assembly workflow**.

Figure S3 **Protein annotation workflow**.

Figure S4 **TEM images of a selection of phage morphologies identified in material from the sampling site**. Siphovirus and myovirus morphologies were observed in particular abundance.

Figure S5 **Multiple sequence alignment of circoviral replication associated proteins (MSA conducted using MAFFT)**.

Figure S6 **Multiple sequence alignment of circoviral capsid-like proteins (MSA conducted using MAFFT)**.
