# Supplementary figures and images for: Virome Assembly and Annotation: A Surprise in the Namib Desert
Source: Front Microbiol. 2017 Jan 23;8:13. doi: 10.3389/fmicb.2017.00013 (PMC5253355; doi:10.3389/fmicb.2017.00013)

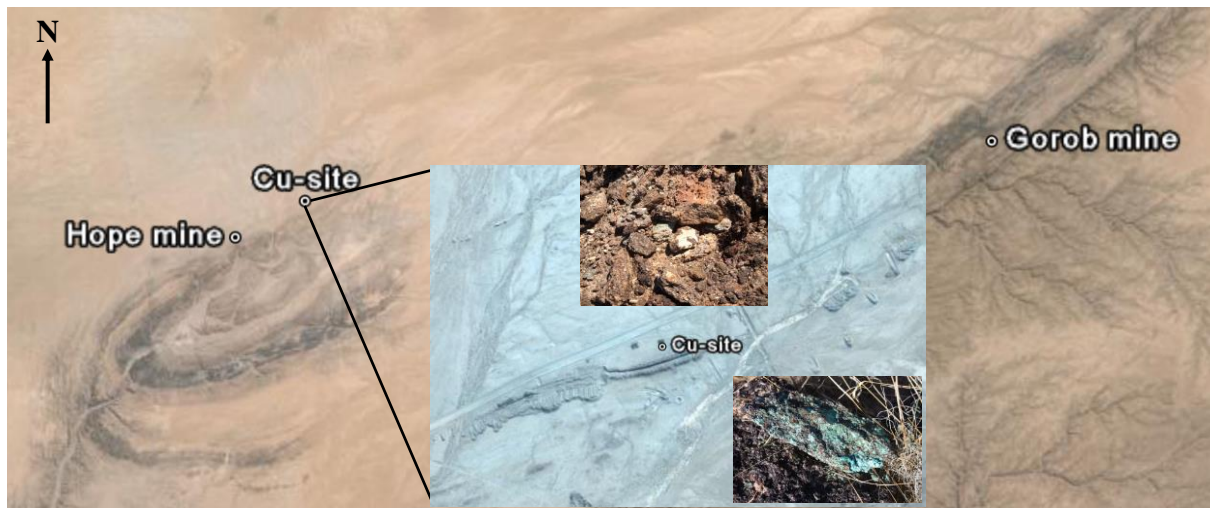

Supplement: Figure S1 — Satellite image of the sampling site (Cu-site) showing its position in relation to nearby copper mines (Hope and Gorob). Inset depicts an enlargement of the site showing the trench dug alongside the road with heaps of sampled copper laden material, as well as images of the sampled material (including material with a green copper patina). [file Image1.PDF]

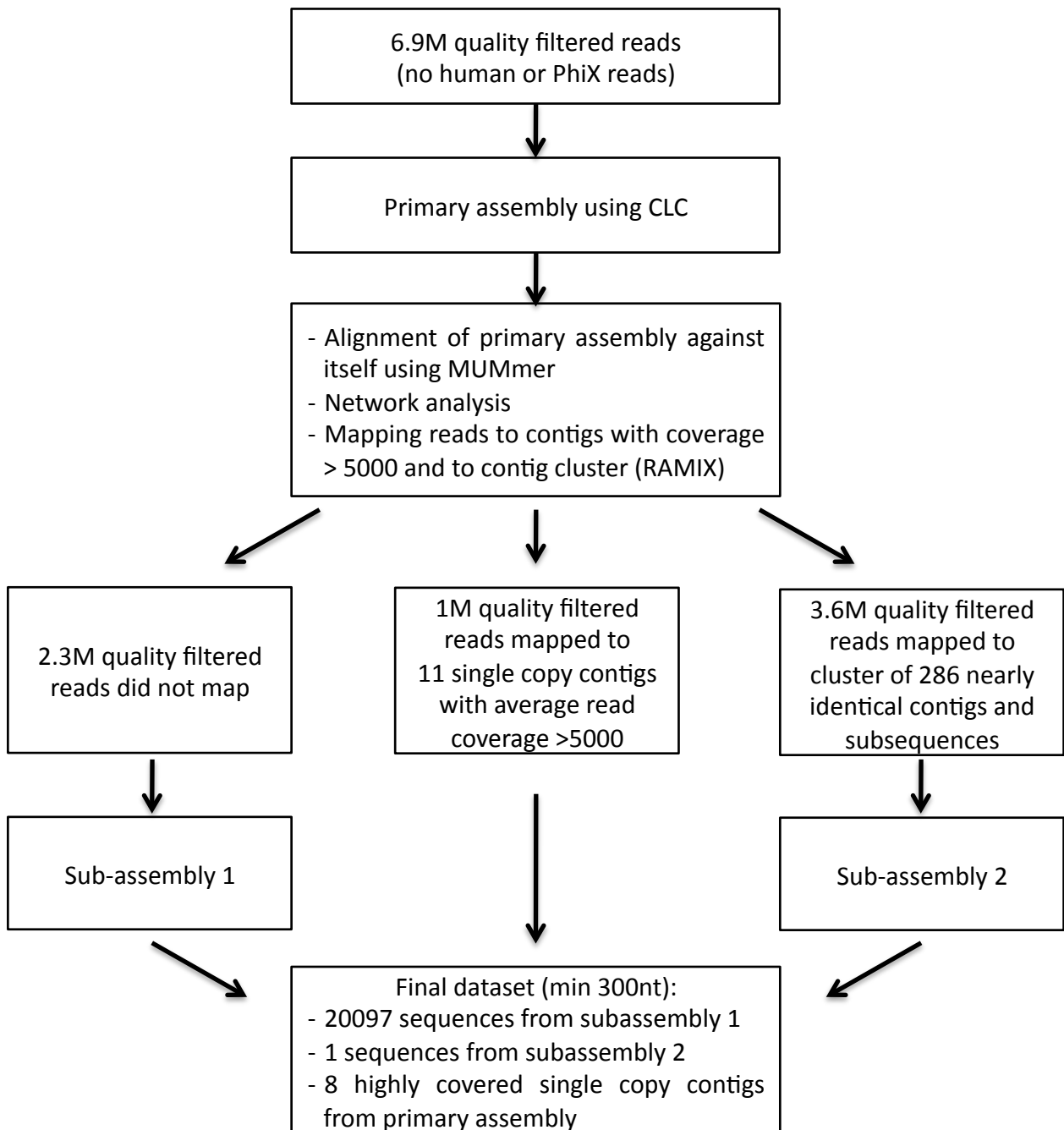

Supplement: Figure S2 — Assembly workflow. [file Image2.PDF]

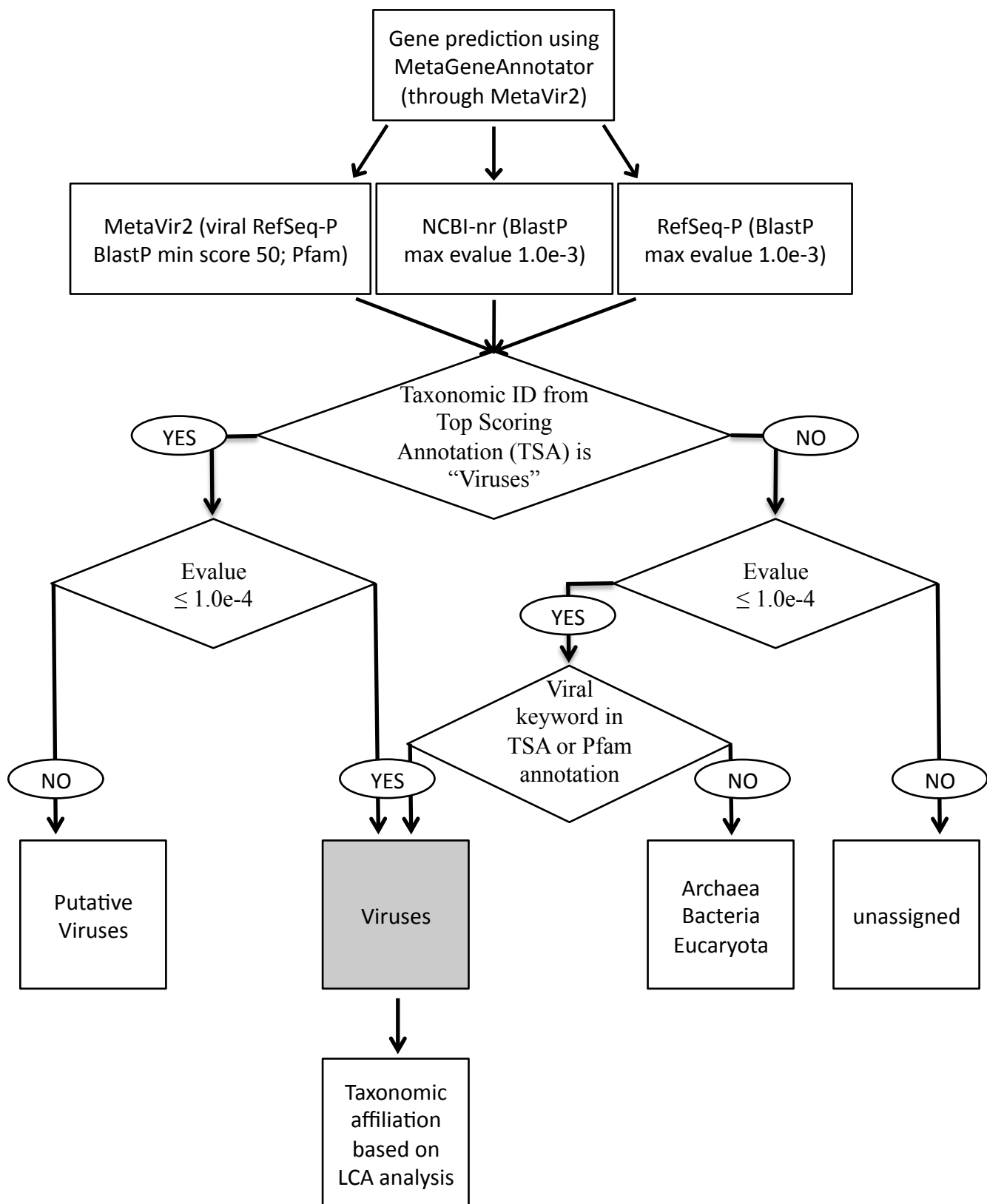

Supplement: Figure S3 — Protein annotation workflow. [file Image3.PDF]

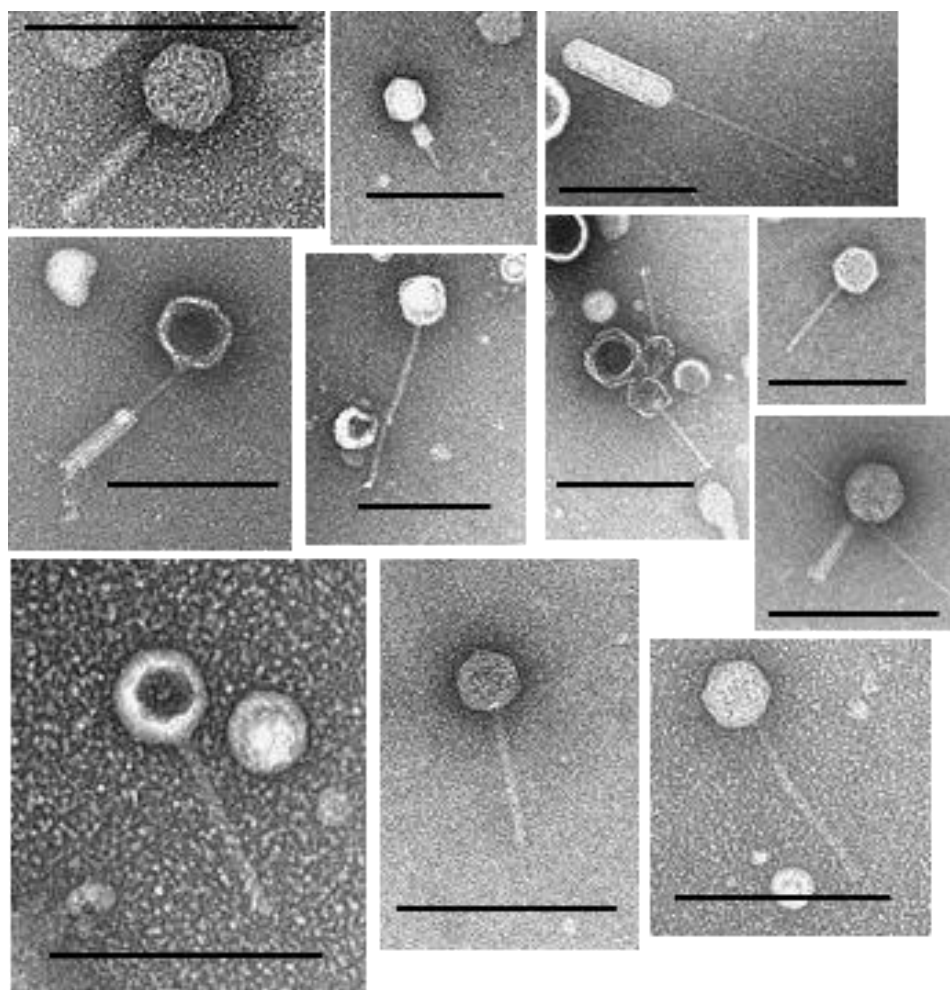

Supplement: Figure S4 — TEM images of a selection of phage morphologies identified in material from the sampling site. Siphovirus and myovirus morphologies were observed in particular abundance. [file Image4.PDF]
