# Supplementary material for: Virome Assembly and Annotation: A Surprise in the Namib Desert
Source: Front Microbiol. 2017 Jan 23;8:13. doi: 10.3389/fmicb.2017.00013 (PMC5253355; doi:10.3389/fmicb.2017.00013)
Supplement: Figure S5 — Multiple sequence alignment of circoviral replication associated proteins (MSA conducted using MAFFT). [file Image5.PDF]

CLUSTAL W (1.8) multiple sequence alignment

```
C_21            MSQNGK------------------------------------------------------
C_71            MEDPHRRSERRELGRSRRPPARYEPVEEPMDDQDQDFDWDQHQSIIDAYLAQRVTTPVVQ
C_5pa           M-----------------------------------------------------------
C_1388          MEASQ-------------------------------------------------------
C_170           M-------------------------------------------ILGCS-----------
Q1L2D4          --MAV-------------------------------------------------------
Q06J75          MPKQA-------------------------------------------------------
D4N3Q0          MAPIK-------------------------------------------------------
Q0H9S9          MPPQK-------------------------------------------------------
Q9IG45          MAPCKPGSNP-------------------------------------------------P
E9NWT8          MAPCKSSTRT-------------------------------------------------P
Q06J78          MRRRP------------------------------------------------------R
A7LI84          M-----------------------------------------------------------
Q8AYV0          M-----------------------------------------------------------
F4YBZ7          MPKAT------------------------------------------------------S
G3LIS4          MPSKK-------------------------------------------------------
B2CR69          MPSKK-------------------------------------------------------
C_351           ------------------------------------------------------------
G1D7G0          M-----------------------------------------------------------
A0A0C5IBG4      ------------------------------------------------------------
S4TE56          M--------------------------------------------------------V--
C_176           ------------------------------------------------------------
922073311       ------------------------------------------------------------
922073300       MP----------------------------------------------------------
922073297       MAH---------------------------------------------------------
A0A0C5IB82      ------------------------------------------------------------
S4TE35          MKREFIFQTCL---------------------------------ILMAF-------QKVF
914343026       MP----------------------------------------------------------
M9LTI9          ------------------------------------------------------------
914343032       ------------------------------------------------------------
M9MJD1          ------------------------------------------------------------
C_2pa           MSKR--------------------------------------------------------
A0A0B4UH75      MDTFPTPNQL----------------------------------ILE---------EDLA
A0A075J175      MEVPPPAYS--------------------------------------------------P
A0A0B4UI50      MEAPPPAYS--------------------------------------------------P
P18921          MSSLPVSESE-----------------------------------------------GEG
A0A0H3XQH5      MAS---------------------------------------------------------
A0A0D5BUM7      MAS---------------------------------------------------------
C_869           MSFPRMTSHRQ-------------------------------------------------


C_21            -----------------------------------------------------------E
C_71            GPTVVSIPADPSDPSPPSVPEEASDSASASGAGSTEPPSGESSCSRNQNTGSNAT----S
C_5pa           -----------------------------------------------------------P
C_1388          ---SVSQPA--SQPASQSVSQSKLDLTKF---------TKHGEREKQ------------T
C_170           --------------------------------------KKKKKNKRK------------V
Q1L2D4          -----------------------------------------------------------R
Q06J75          -----------------------------------------------------------R
D4N3Q0          -----------------------------------------------------------R
Q0H9S9          -----------------------------------------------------------R
Q9IG45          KGRVSAAE------------------------------GGARREATR---------RPPR
E9NWT8          PGRVSAAE------------------------------GGARREATR---------RPPR
Q06J78          SGRYLRSI-----------------------------------MAAR------------R
A7LI84          --------------------------------------------AKS------------G
Q8AYV0          --------------------------------------------AKN------------G
F4YBZ7          KTRHLQNP---------------------------------RQDAPR------------R
G3LIS4          ----------------------------------------------S------------G
B2CR69          -------------------------------------------NGRS------------G
C_351           ------------------------------------------------------------
G1D7G0          -----------------------------------------------------------P
A0A0C5IBG4      ------------------------------------------------------------
S4TE56          -----------------------------------------------------------P
C_176           ------------------------------------------------------------
922073311       ------------------------------------------------------------
922073300       -----------------------------------------------------------R
922073297       -----------------------------------------------------------R
A0A0C5IB82      ------------------------------------------------------------
S4TE35          HPASSSSTSE-DDSSTQTVTRAPTRTSHIAISGD----SGGSTAARNSGASAAATQTHGS
914343026       ------------------------------------------------------------
M9LTI9          ------------------------------------------------------------
914343032       ------------------------------------------------------------
M9MJD1          ------------------------------------------------------------
C_2pa           --------------------------------------------KRT------------P
A0A0B4UH75      EPETLEAPTE-------------------------------KATAKG------------F
A0A075J175      PPAAVSAEV----------------------------------VPRP------------F
A0A0B4UI50      PPPSVTPPIE---------------------------------SSRP------------F
P18921          SGTSVQVPSRGGQ---------------------------VTPGEKA------------F
A0A0H3XQH5      --------------------------------------------NSN------------F
A0A0D5BUM7      --------------------------------------------SSN------------F
C_869           --------------------------------------KKRKISSRKHGAGGNTL--PPR


C_21            PKSSRNVCWTWNNPPGVGLDMSETEYAAYAQAAMQQLS-SMSGCNFVVFQLERG--EQG-
C_71            SRRCRNWGYTSWQDT-----------------SLPTE--LPPGVRYICAQQEIAPTTTG-
C_5pa           RITSRNFILTIFNEE------------------LLPTS-ETPGLRYCVYQKEKCP-TTA-
C_1388          NHAAKNWVFT------------------DVT-DLSPFGELPSGVKYTISQVEKAP-TTG-
C_170           RIAAKYWCFTLNNYS------------------TPPWDAKPKGVTYMVCQAEKGE-KEG-
Q1L2D4          GSAAKRWCFTLNNPT-----------EEEIA-AVKAW--QHSEYHYAIVGKEKG--EQG-
Q06J75          ESPCKRWCFTLNNPT-----------EEEIE-RVKNL--SPSEYHYAIVGKEKG--EQG-
D4N3Q0          PAPCKRWCFTLNNFT-----------DDEVQ-KVVSL--QPDEVHYAIVGRETG--AQG-
Q0H9S9          EAAAKRWCFTLNNYT-----------DEEVS-AVKAW--NASEYHYAVVGREKG--ENG-
Q9IG45          EAAAKRWCFTLNNPT-----------EEEIK-SLETW--LVSDFHYAIVGKEVG--EQG-
E9NWT8          EAAAKRWCFTLNNPT-----------EDEIK-SLDSW--LLSEFHYAIVGKEVG--EQG-
```

```
Q06J78          DSGARRWCFTLNNYT-----------PEEEE-TARNLIHDADKYAFAIIGKEVG--ESG-
A7LI84          NYSYKRWVFTINNPT-----------FEDYV-HVLEFC-TLDNCKFAIVGEEKG--ANG-
Q8AYV0          NYSYKRWVFTINNPT-----------FEDYC-SVVEFC-NLDNCKFAIVGEEKGE-KEG-
F4YBZ7          EQPVKRWCFTLNNPT-----------AEERR-HIQEII-TADAVDFAVIGNEVG--DSG-
G3LIS4          PQPHKRWVFTLNNPS-----------EEEKN-KIREL--PISLFDYFVCGEEGL--EEGR
B2CR69          PQPHKRWVFTLNNPS-----------EDERK-KIRDL--PISLFDYFIVGEEGN--EEGR
C_351           -MKGRYWLLTIPYNE------------------FTPY--LPKPACFIKGQLETGN-TTG-
G1D7G0          CLQARYWLLTIPYEH------------------FTPY--LPPNCAYIKGQLEQGS-NTS-
A0A0C5IBG4      MNQARFWILTIRHAD------------------FLPY--LPPTVDYLRGQLERG--DGG-
S4TE56          IPQARYWLLTIPHAE------------------FVPY--LPPGFNWIRGQLELG--AEG-
C_176           -MQGKFWCFTINNPT-----------AADVT-TLSSLV-ESGEASYLVFGRETAP-TTG-
922073311       -MTSCRYCFTVNNPT-----------VQDRE-RLDLLA---DSCNYLVYGNEIG--SSG-
922073300       DAPAKQWCFTLNNYT-----------PAELT-AIVD---SAGNFDYLCFGRERG--NNN-
922073297       QHAAKRWVFTINNWT-----------AAEQQ-ALID---SSDNFDYLCFGRERG--DNN-
A0A0C5IB82      MAQSRSWCFTLNNYV-----------QADID-RLAAFG-ETDDCTYLVFGKEVG--ESG-
S4TE35          GGRAKNWCFTLNNYT-----------DDEEQ-CIGDFS-ENDHLLYMIVGREKG--DSG-
914343026       TFQSARWCFTVNNPT-----------DADAS-AIAELG-DGPLTRYLVVGREIG--ESG-
M9LTI9          MPGGKHWVFTVNNYD-----------EQTLL-HVQLLS-ESDAVTYCVIGREIAP-TTG-
914343032       MSRAKNWVYTLNNPT-----------GDEIS-KAEGLFLSTPGVIYHVFGKETG--DSG-
M9MJD1          MAQSKHWCFTINNWN-----------AEDDE-RLQELG---NNCEYLVYGYETG--AQG-
C_2pa           LTKAKNLFLTFPQNS---------TTKEQLMTQIKGH--FGDNLDYTIVCEEKH--KDE-
A0A0B4UH75      RLSGKNFTITFPQCD---------VKKEIAVERIEQK--FGSEIKGYLVCEEQH--KDG-
A0A075J175      RLAGKAIFLTWPQND---------VSKEDLMAKIVAL--WEAKLSWAVVAEESH--KSG-
A0A0B4UI50      RLAGKAIFLTWPQND---------ITKEDLMAKLVSL--WEAKLSWAVVAEESH--KSG-
P18921          SLRTKHVFLTYPRCP---------ISPEEAGQKIADRL-KNKKCNYIYISREFH--ADG-
A0A0H3XQH5      RLAAKNVFLTFPQCP---------ESITWVMSHLLQIL-NSYTVKYACVAEEKH--KDD-
A0A0D5BUM7      RLAAKNVFLTFPQCP---------ESITWVMSHLLQIL-NSYTVKYACVAEEKH--KDD-
C_869           EVRSKRWTFTLWDPD-----------VTDIT-SLDTVL---KTYEY-IFQVEVG--KTG-


C_21            TLHYQGYSEFTKGVKWTAFKSATG------VQLHCE--ARR-GSQDSAIDYCCKEEGRIA
C_71            ELHVQGFMQLETPKSLSAMKKKYG------RTLNFQ--EHRYGSYQAMRDYCMKERTRAP
C_5pa           RVHWQTFLQYKRAIRLTTARKRF-------PSTDVR--IAE--NPPAARLYCMKEESRLE
C_1388          KLHRQGYVQLTVRKRLSFLSTLR-------PHAYWS--RAR-GSCQQNQIYCSKADTRHE
C_170           TIHLQGYIQFDRSRELSWIRRVVS------DRAHWE--VMRAHNSDAARDYCMKEDTRIG
Q1L2D4          TPHLQGFIHLKKKVRLTSLKKVL-------QRAHWE--KAR-GSDEDNEKYCSKEGDVI-
Q06J75          TPHLQGFLHLKKKQRLKQMKELI-------PRAHFE--RAR-GSDEDNEQYCGKEGDVI-
D4N3Q0          TPHLQGYLHLKKKKRLTSMKEFL-------PRAHWE--VAR-GSDEDNEAYCSKEGDVI-
Q0H9S9          TPHLQGYIHLKKKARLSTLKKLL-------SRAHWE--KAR-GSDSDNEAYCTKDGDVI-
Q9IG45          TPHLQGFVHLKQKKRLPQLKQLF-------KRAHWE--KAR-GSDEDNEKYCSKEGNVL-
E9NWT8          TPHLQGFIHLKQKKRLSQLKQLF-------NRAHWE--KAR-GSDEDNEKYCSKEGNVL-
Q06J78          TPHLQGFMHFKQKQRLTALKKLF-------PRAHFE--KAR-GSDQQNADYCGKDGEIL-
A7LI84          TPHLQGFLNLRSNARAAALEESLG------GRAWLS--RAR-GSDEDNEEYCAKESTYL-
Q8AYV0          TPHLQGFLSLRKNAKAAALEEKLG------GRAWLS--RAR-GSDEDNEEYCSKESTYL-
F4YBZ7          TPHLQGFLNMKTKRRLGTMKKWFN------ARAHYE--AAK-GTDLQNDEYCTKGGDTY-
G3LIS4          TPHLQGFANFAKKQTFNKVKWYFG------ARCHIE--KAK-GTDQQNKEYCSKEGHIL-
B2CR69          TPHLQGFANFVKKQTFNKVKWYLG------ARCHIE--KAK-GTDQQNKEYCSKEGNLL-
C_351           YLHWQLIVYFEKQQRLAALKKIFG------EGIHAE--PTR---SEAVEQYVFKDETRVD
G1D7G0          YLHWQLVVYFSQKKSLNYVKLIFG------DGIHCE--PSK---SKAAEEYVWKEDTSIP
A0A0C5IBG4      FLHWQLVVHFARKVRLGGVKGIFG------DSTHAE--PTR---SDAAREYVWKEDSAVP
S4TE56          YLHWQIFVSCVQKSRRRGITSVFG------TGIHAE--PSR---SAAAEDYVWKEDTRVE
C_176           TPHLQGYVAFVSNQRRAAATTKLG------GRASVR--LKR-GTHKQASDYCKKDGDFE-
922073311       TPHLQGFVIFPKTKRFNAAKIAIG------NTAHVE--CAR-GSSVQAATYCKKDGDFR-
922073300       TPHLQGYLILKEKKRFSYVRQLAG-----LERAHWEKKSPR-STPKQASDYCKKDGDYD-
922073297       TPHLQGYVILKTKLRLNNVKALPG-----FRRCHLE--VSR-GTPQQAADYCKKDGDFE-
A0A0C5IB82      TPHLQGFAIFPRKLRLRAVKSHIG------NGAHLE--CAR-GSPLQASDYCKKDGDFT-
S4TE35          TPHLQGYLSLNRRFTLSQIKGWLG------SRVHLE--VAR-GSPQQNRDYCTKEGDYD-
914343026       TRHLQGFVIFHQVQSRAAVSGYI-------ARAHLE--PAR-ASSVQASDYCKKDGDFD-
M9LTI9          TPHLQGYLSLSTRKSFAAVRDML-------PACHIE--GAR-GTPSQCRTYCIKDGDYD-
914343032       TFHLQGYVIFSQRKRLGQLREIF-------PRGHFE--VSR-GSPHQASDYCKKEGDFK-
M9MJD1          TPHLQGYVVFRTKVRMGIARGLLG------GRAHVE--IKR-GTPLQAATYCKKEGLFF-
C_2pa           GDHLHAFIALKKQLRVYWKDLDKF------TGKHGNY-QAA-RNKSKSVKYCMKDGSWI-
A0A0B4UH75      TPHLHVYLSFLNKKNFKCHHCFNF-----IGGKQGNY-QVT-KSVRDWVTYCTKGDNYV-
A0A075J175      EPHVHAIVQFAERLDLKNANPVLD----ALTGKHGNY-QSV-KSAKKVLRYVCKDGQYI-
A0A0B4UI50      EPHVHAIAQFTERIDLKNANPVLD----ALTGKHGNY-QSV-KSAKKVLRYVCKDGQYI-
P18921          EPHLHAFVQLEANFRTTSPKYFDL------DEFHPNI-QAA-RQPASTLKYCMKHPESS-
A0A0H3XQH5      NSHFHAIVQLDKKLETRNPNFFDLPQEHSSGTYHPNV-QKC-SSPAAVRKYIQKEGHFI-
A0A0D5BUM7      NSHFHAIVQLDKKLETRNPNFFDLPQEHSSGTYHPNV-QKC-SSPAAVRKYIQKEGHFI-
C_869           RQHLQGYIEHSEAIRFSTLKSLFK------DGTHLE--TAK-GSRDQNIKYCTKPNGKRS


C_21            --GPFRSGEPRCNR---------------PGLRTDIQQFRDD-----IR-AG-----K--
C_71            GTEPVEFGDFKK-----------------PGQRSDLREFVEE-----VK-SG-----K--
C_5pa           --PPVEIGTFLT-----------------QGARTDIVTFRDA-----IQ-GG-----K--
C_1388          DGAPEELGTPIT-----------------KGQRTDLEAFYQD-----IK-SG-----QY-
C_170           --GPWEWGTYTNVKGGYGRGRRRKALEGGGGRRTDIEAFRDA-----IL-GG-----M--
Q1L2D4          ----LTIGIPV------------------KGNRSDLAGAVAA-----VK-AG-----R--
Q06J75          ----LTIGVPS------------------KGNRSDLAGAVAA-----VK-AG-----R--
D4N3Q0          ----LTLGIPA------------------KGNRSDLSGAVAA-----VK-AG-----R--
Q0H9S9          ----LTLGMPV------------------EGNRSDLSGAVAA-----VK-AG-----S--
Q9IG45          ----LTLGIPA------------------KGNRSDLSEAVAA-----VK-AG-----R--
E9NWT8          ----LTLGIPA------------------RGNRSDLSGAVAA-----VK-AG-----R--
Q06J78          ----TMIGTPS------------------DNNPSDLAGAVAA-----VK-RG-----S--
A7LI84          -----RVGEPVS-----------------KGRSSDLAEATSA-----VM-AG-----V--
Q8AYV0          -----RVGEPNR-----------------KGRSSDLSDAASD-----VL-AG-----L--
F4YBZ7          ----LRIGEPGK-----------------ERCRNDLQKAIDV-----VKRSS-----G--
G3LIS4          ----IECGAPRN-----------------QGKRSDLSTAVST-----LL-ET-----G--
B2CR69          ----MECGAPRS-----------------QGQRSDLSTAVST-----LL-ES-----G--
C_351           G-TQFELGKRAI-N---------------RNNPKDWEAILSS-----AK-QG--------
G1D7G0          G-TQFELGKNSI-K---------------RDSTKDWDLIVKH-----AR-EG--------
A0A0C5IBG4      N-TRFELGKIPV-R---------------RGVSHDWDAVRES-----AK-RG--------
S4TE56          G-TQFELGTKPV-N---------------RNSPADWTAIRTS-----AV-AG--------
C_176           -----EYGELPTEQ---------------QGKRSDWDRYKEW-----VV-EL-----GRI
922073311       -----EFGELPS-S---------------QGKRTDWDIYRDW-----VT-DL-----GRV
922073300       -----EYGELPTKK---------------QGQRTDFDELKEW-----IK-EQ-----DHR
922073297       -----EFGELPK-G---------------QGKRSDFENLKEW-----IK-SL-----DHW
```

```
A0A0C5IB82      -----EFGSFGGVR---------------PGLSGRFGQFVEW-----LD-EHYKEGNVHQ
S4TE35          -----EYGILPRTT---------------QGKRNDFICYQQW-----VV-SL-----DRM
914343026       -----EYGVCPR-----------------SGQRFDLQALLKWGD-DFIA-SH-----KRA
M9LTI9          -----EYGTLPDVQ---------------QGKRTDFERFRDW-----CS-DR-----SCV
914343032       -----EFGSLPPQA---------------QGKRTDWERLREY-----VE-QL-----GRR
M9MJD1          -----ETGELPG-P---------------QGVRNDMVHARDW-----II-RHQEE-HGDV
C_2pa           -----AHGFEPESY-----------IKAASGRKSTKATIVAQ----EII-----------
A0A0B4UH75      -----AKGLDVQ---------------AIKKKTAPKSTTVAT----MLM-EG-----K--
A0A075J175      -----THGEVPD-----------------FSEKPKLQDWAAK----LIIEEQ-----K--
A0A0B4UI50      -----THGEVPD-----------------FAEKAKLQDWAAK----LIMEEQ-----A--
P18921          ----WEFGKFLK----------------PKVNRSPTQSASRDKTMKQIMANA----TSRD
A0A0H3XQH5      -----EHGEFNT-RGRSPVASAEKIFGEILTLATDEESFLAL-----VR-ER--------
A0A0D5BUM7      -----EHGEFNT-RGRSPVASAEKIFGEILTLATDEESFLAL-----VR-ER--------
C_869           A----TYSNMLPESGFD------------ITRMTDWQRAPPP-----AS-GG-----CEV


C_21            -RRHELEDSHPLLCAKYPKFISFVR-NGT-------------------------------
C_71            -RKLDLLESHTSIIARYPKFYKDIR-ELY-------------------------------
C_5pa           -RKRDLVESHPLMLAKHAKFYDLVT-SLN-------------------------------
C_1388          -SKSEIFDLHPGQMIRHRHAYDDIR-ALTK------------------------------
C_170           -GNLQLWQEFPAQMAKYPRMYAQLH-SCP-------------------------------
Q1L2D4          -GMSEIAREFSETYVRYGRGLRDLA-LLI-----------------GQ------------
Q06J75          -GMAEVAREFSLAYVRYGRGLRDLA-LLI-----------------GQ------------
D4N3Q0          -QMREIARDFSEVYVKYGRGLRELA-LLI-----------------GQ------------
Q0H9S9          -RMVDIAREFSEVYVKYGRGLRELA-LLI-----------------GQ------------
Q9IG45          -AMTEVARDFSEIYVKYGRGLRDLK-LLI-----------------GQ------------
E9NWT8          -AMTEVAREFSEIYVKYGRGLRDLK-LLI-----------------GQ------------
Q06J78          -QMSEIAREFSEVYVKYGRGLRDLR-LLI-----------------GC------------
A7LI84          -PLTEVARKFPTTYVIFGRGLERLR-HLI-----------------VE------------
Q8AYV0          -PITDVARKYPTTYVMFGRGLERLR-QLI-----------------VE------------
F4YBZ7          -SMRAVAEACPATFIRYGRGLRDYA-NVMQ----------------YR------------
G3LIS4          -SLVTVAEQFPVTYVRNFRGLAELL-KVS-----------------GKM-----------
B2CR69          -SLVTVAEQHPVTFVRNFRGLAELL-KVS-----------------GKM-----------
C_351           ----RLDDIPPDVYIRCYNSLKRIAVDNL-------------------------------
G1D7G0          ----DFASIPGDVLVRCYGNLKKIRVDSL-------------------------------
A0A0C5IBG4      ----RLDDIPADIYCRLYGNFKRIAVDHM-------------------------------
S4TE56          ----DLAGVPSDVYVRCYHQLRSIGKDHL-------------------------------
C_176           PTRDELINHNTSLYARYSKRCYEIA-AAY-----------------LPSPQL-VAPNQ--
922073311       PSKKELVLAFPGFYARYRKACFEYA-EAL-----------------TPPPIL-TQS----
922073300       PTDREVAEEFPSLWGRYRSACISFL-DLF-----------------SPHPTL-VQG----
922073297       PDDHEIAEEYPSLWGRYRSACESFR-QLF-----------------GKPIEV-VDPTT--
A0A0C5IB82      PSRALIAATWPDLYVRYHTKLFELV-GVL-----------------APKPIL-QEG----
S4TE35          PTMHEIAREWPGLFARYSDRLRTIA-EAS-----------------LPPIDL-LGDSY--
914343026       PTAHECAVEQPAAYLKYPR-LISLF-QAR-----------------APPPDFGREG----
M9LTI9          PSNRTLMLEWPSLYGRYRSAMRAMA-DEL-----------------SPALEL-RTG----
914343032       PLERDLILEFPNLVG----ASYNIS-HDL-----------------HPQKRV-ANGSGK-
M9MJD1          PTELELIMEIPGLYGRYRSNLLSMC-HAL-----------------RKKPEF-DTG----
C_2pa           ------------------------------------------------------------
A0A0B4UH75      -SLSEINAVDPGYVMINKRKLEEYE-SWVTIERNKKSKLTWVP---PLLDGL-TDANKQI
A0A075J175      -TYRDLVREQPGFSMMQKRKLEEFI-GWNKRQKLADSLLPW--RVLTPKPSA-SPVHVAL
A0A0B4UI50      -TYRDLVRQQPGYCMLQKPKLEEFI-GWSKRQRLADSLLPW--RVLTVKPNA-PYVHQAL
P18921          EYLSMVRKSFPFEWAVRLQQFQYSA-NALFPDPPQTYSAPYASRDMSDHPVI--------
A0A0H3XQH5      -RPQDYVLRWPSITG-------------FARDHYRRRSIPYVPRW-TDFPGL-PEPIQQ-
A0A0D5BUM7      -RPQDYVLRWPSITG-------------FARDHYRRRSIPYVPRW-TDFPGL-PEPIQQ-
C_869           DTLGGASPHTPVAVATTPTSGSVLD-AVT-------------------------------


C_21            ----------RSS---------------FRRKDVVLLVGSPG-VGKTRFAEEQGG-GDLW
C_71            ----------PPK---------------RDFKGNCLIIGPTG-CGKTTFARTNFE-NDMY
C_5pa           ----------RPA---------------RKDRQVLLITGPTG-AGKTRMVYDQWKDEDWY
C_1388          ----------KPT---------------NPDKQVYLLYGPTG-LGKTRLVHEACKDEGLW
C_170           ----------KVR----------------DPPTIILHYGPTG-CGKTKWFFDNCPDDDWY
Q1L2D4          ----------KQR---------------DFKTEVTVLTGPSG-VGKSRWANEQVG--TKF
Q06J75          ----------KPR---------------DFKTEVIVLTGPSG-CGKSRWANEQEG--TKF
D4N3Q0          ----------KPR---------------DFKTEVIVLTGPSG-VGKSRWANEQEG--AKF
Q0H9S9          ----------KPR---------------DFKTEFIVVTGPSG-VGKSRYANEYPG--TKF
Q9IG45          ----------QPR---------------DFKTEVIVITGPPG-CGKSRWAADYPG--SKF
E9NWT8          ----------QPR---------------DFKTEVIVITGPPG-CGKSRWAAEYPG--SKF
Q06J78          ----------PPR---------------DFKTEVIVLIGPPG-CGKSKLANEMEG--SKF
A7LI84          ----------TQR---------------DWKTEVIVLIGPPG-TGKSRYAFEFPA-ENKY
Q8AYV0          ----------TAR---------------DWKTEVIVLIGRPG-SGKSRYAFEFPA-REKY
F4YBZ7          ----------KPR---------------DFKTEVNVYVGDPG-CGKSRKASELCAGTTVY
G3LIS4          ----------QQR---------------DWKTAVHVIVGPPG-CGKSQWARNFAEPSDTY
B2CR69          ----------QKR---------------DWKTNVHVIVGPPG-CGKSKWAANFADPETTY
C_351           ----------KPC---------------EIEREIKVYCGRTG-TGKSRRAWEEAG-ISAY
G1D7G0          ----------QPE---------------SIVREIYVYYGRTG-AGKSRRAWEEAT-LSAY
A0A0C5IBG4      ----------VPL---------------GIEREVNVYWGVTG-SGKSRRAWNEAG-LDAF
S4TE56          ----------QPV---------------AMVRTCNVFWGRTE-TGKSRRAWEEAG-LEAF
C_176           ----------QPRFGWQLRVEGLITAENASDRSIHFVVDPEGNSGKS-------------
922073311       ----------EPRFGWQTRVDGIINGE-ANDRTIHFVVDPEGNAGKT-------------
922073300       ----------TLR-PWQEDLNTRLSAP-PNDRDVMFVVDENGNSGKS-------------
922073297       ---------FEPR-VWQQRIIDICNGE-PDPRKVYFVVDENGNTGKS-------------
A0A0C5IB82      ----------DPN-AWQTTLIEALGLD-PDDRKIYFYVDETGGSGKS-------------
S4TE35          ----------ELR-PWQAELASSLEDP-PDPREVMFFVDSDGGKGKS-------------
914343026       ----------ERR-PWQHELEDELEGRCENDRRVTFIVDQVGGAGKT-------------
M9LTI9          ----------ELR-PWQTSLFTRLNEA-ADDRTVEFYVDANGGSGKS-------------
914343032       --------P-TPRI--------------PRQPAIEFIVDPEGNSGKT-------------
M9MJD1          ----------EMR-PWQAELNEILKLE-PDDRKILFYVDAIGGAGKS-------------
C_2pa           -------------------------------------------AGKS-------------
A0A0B4UH75      CEWICSNIR-QPR---------------KFKAPQLFITGPKN-LGKTSLIEWLGQYLSLY
A0A075J175      WTFLRENVL-VPR---------------TPRQAQLWLCGLPG-VGKTRFLAYLRARLRVY
A0A0B4UI50      WTFLRENVL-VPR---------------TPRQAQLWLSGLPG-VGKTRFLSYLRARLRVY
P18921          ------------------------------------------------------------
A0A0H3XQH5      --WAKDNILFVSR----------------------------------QWADSV-------
A0A0D5BUM7      --WAKDNILFEPE-------------SKPDRPISLYICGPTR-SGKTQWDRSLGR--HNY
C_869           ----------EAE---------------EEKRRIHWYWEPNGGCGKTRLARHLAI-----
```

```
C_21            VQPIGG-QGW-FDGY----------------------------DMQKHALFDDFMG-KGS
C_71            VMPCGK-SLW-FDGY----------------------------EGQEIVLMDDFSG----
C_5pa           EVPLDE-NIW-FDGY----------------------------DSHTHVLIDDFVG----
C_1388          EPYIGN-AKW-FDGY----------------------------DQEPNVLLDDFDG-ALS
C_170           STPISNGTLW-LDGY----------------------------REQSWVLFDDFSG----
Q1L2D4          YK--MK-GDW-WDGY----------------------------CNEDIVIIDDFYG----
Q06J75          YK--MK-GDW-WDGY----------------------------SNEDMVIIDDFYG----
D4N3Q0          YK--MK-GDW-WDGY----------------------------SNEDIVVIDDFYG----
Q0H9S9          YK--MK-GDW-WDGY----------------------------SNEDVVVIDDFYG----
Q9IG45          YK--MK-GEW-WDGY----------------------------DHQEVVIIDDFYG----
E9NWT8          YK--MK-GEW-WDGY----------------------------DHQDVVVIDDFYG----
Q06J78          YK--MK-GDW-WDGY----------------------------DNQDIVIIDDFYG----
A7LI84          YK--PR-GKW-WDGY----------------------------SGNDVVVMDDFYG----
Q8AYV0          YK--SR-GKW-WDGY----------------------------NGQDVVVMDDFYG----
F4YBZ7          YK--PR-GMW-WDGY----------------------------DGQENVIVDDFYG----
G3LIS4          WKP-SR-NKW-WDGY----------------------------HGEEVVVLDDFYG----
B2CR69          WKP-PR-NKW-WDGY----------------------------HGEEVVVIDDFYG----
C_351           PKD-PR-TKF-WDGY----------------------------GGQTNVVMDEYRG----
G1D7G0          PKD-PN-TKF-WDGY----------------------------AGQECVVIDEFRG----
A0A0C5IBG4      PKD-PR-TKF-WDGY----------------------------RGHENVVIDEFRG----
S4TE56          PKN-PR-SKF-WDGY----------------------------RDHEHVVMDEFRG----
C_176           ---------W-MCRWALSKHPNKVQVMRIGKRDDLAYSV---DETKSVFLFDVPRN-QMT
922073311       ---------W-FCSYALTKWPDKVQVMRIGKRDDLAYAI---STEKSIFLMDVPRN-QMT
922073300       ---------W-FIRYLMTERPDDVQMLSIGKRDDLAHAI---DPAKKIFFFDVPRG-GME
922073297       ---------Y-LSAYLISKFPDEVQILSVGRRDDLAHAI---NPRRSIFLFDVPRG-GME
A0A0C5IB82      ---------W-LTRYLMTSR-DDVQALSIGKRDDIAHAI---DVTKKVFLFNVPRT-QME
S4TE35          ---------Y-FCQWLLSKSTDG-QVLRIGKRDDLAYAI---DETKKIFVFDIPRT-QME
914343026       ---------W-FQQWFLSKNPARAQIVSIGKRDDVAHTI---DETKEVFFFAVPRG-QME
M9LTI9          ---------W-FCGYMVSK--PGVQLLGPGKRDDLAYMI---DATKHIFLLNVPRQ-QME
914343032       ---------W-FCQYMITQHPDSVQYLRIAKRDDLTFAI---DETRTIFLFDVPRG-QME
M9MJD1          ---------W-YQRYKLTTC-NDVQLLSSGKRDDIAHAV---DEQKRIFFFNVPRG-GMQ
C_2pa           -------------NYDLAKDPDG-------------------------------------
A0A0B4UH75      HIP-QT-EEF-YDLY---------------------------TDDYDLVVFDEFKG----
A0A075J175      DMP-RS-EDF-YDEY-----------------ED---------GCFDLVVLDEYKA----
A0A0B4UI50      DMP-RD-EDF-YDDF-----------------ED---------GCFDLVVLDEFKA----
P18921          -------GEW-LQQELYTVSPQALS-LHAG--------------------ISEEQA----
A0A0H3XQH5      ---------LCYRCH-----------------DDF-------------------------
A0A0D5BUM7      FT-----GSLSFLDY-----------------DDFAL----------YNVIDDIEYEKIS
C_869           ---------YYSAAY-----------VSGKAHDIKYAMKEHHEDATIVVWDLKRC---M


C_21            KMRLDDLLRLLDGYSVQVPVK--GDFVPWCPPETIYITSNYHPREWYD-WKDRLPSYYAL
C_71            QYPLSALLQLLDRWTQRVEIK--GSHTWFNPM-GILVTTNIHPQSWYD-FRGREEQRNAL
C_5pa           QYKLVLLLRLLHAYPERVPVK--YGYVWWNPE-KIIITSNFEPRKWYRNWEDREPHYHAL
C_1388          HYSLKTTLKLLDIYRTRVEIK--GGGLHFQPD-TIYITTNIHPQNWYK-WEGREAQRPAL
C_170           QMPLVQLLRMLDRYPITVPIK--GSHTDFSPAKTIVITTNIHPAKWYK-WSNRKLQYDAL
Q1L2D4          WIPFCELLRLCDRYPHKVPVK--GSYVEFNSK-QIIITSNTPPDSWYN-EDK--CYVQAL
Q06J75          WIPFCELLRLCDRYPHKVPVK--GSYVEFTSK-KIIITSNTHPDHWYN-EEK--CYLQAL
D4N3Q0          WIPFCELLRLTDRYPHKVPVK--GAYVEFTSK-KIIITSNTPPESWYN-EEK--CYVQAL
Q0H9S9          WIPFCELLRLTDRYPHKVPVK--GSYVEFTSK-VIIVTSNTHPDSWYN-EEK--CYLPAL
Q9IG45          WLPFCELLRVTDRYPHKVPVK--GAFVEFTSR-VIIVTSNSPPDAWYS-EER--CCVQAL
E9NWT8          WLPFCELLRVTDRYPHKVPVK--GAFVEFTSR-VIIVTSNSPPDAWYS-EEK--CCVQAL
Q06J78          WLPYCECLRLCDRYPHRVPVK--GAYVEFTSK-KIVFTSNRHVDGWWK-GE---IEKSAF
A7LI84          WLPYDDLLRITDRYPLRVEFK--GGMTQFVAK-TLIITSNREPRDWYK-SE---FDLSAL
Q8AYV0          WLPYDDLLRICDRYPLRVEYK--GGMTQFVAK-TLIITSNREPRDWYK-CE---FDVSAL
F4YBZ7          WMPCDELLRVFDRYPCKVPVK--GAYVEFVST-NIYVTSNKHVWQWYK-FEG--FDPAAV
G3LIS4          WLPWDDLLRLCDRYPLTVETK--GGTVPFLAR-SILITSNQAPQEWYS-STAV-PAVEAL
B2CR69          WMPWDDLLRLCDRYPLTVETK--GGTVPFLAR-SILITSNQTPLEWYS-STAV-PAVEAL
C_351           AIDISHLLRWLDRYPVIVEVK--GGATVLQAK-YIWITSNLHPKDWYP-DLDP-ETLQAL
G1D7G0          AISISHLLRWLDRYPVIVEIK--GSSCVFKAK-KIWITSNLSPDDWYP-DLDA-ETKSAL
A0A0C5IBG4      GIDVAHLLRWFDRYPVVVEVK--GSSVVLSAK-HIWITSNLDPREWYA-DLDA-ETLAAL
S4TE56          AIDISYLLQWLDRYPVIVEVK--GSSVVLRAR-TFWITSNLDPRLWYP-DADA-ETMSAL
C_176           YLQYSVLESLKDQVIFSPKYE--SSCKILRTKPQVIIFSNEHPD-----------MNALT
922073311       FLQYSVLEMLKDRMIFSPKYE--SSFKILQYVPHVIVFSNEQPD-----------TSALS
922073300       FMQYAVLEQLKNRLVFSPKYE--SRMKVLHHIPHVVVFCNEEPD-----------RTKLS
922073297       YLQYTIFEQLKNRTVFSPKYN--SITKILRKLPHVIIFSNESPD-----------RNKMS
A0A0C5IB82      FLQYSILESLKDRMVMSPKYN--SMMKVLHSVPHVVVFSNEEPD-----------RTKLT
S4TE35          YLQYSVLEMLKDRMVFSAKYQ--SATKILRQSPHVIVFSNEYPD-----------MSKMS
914343026       FLRYEILEMLKDRMVFSPKYA--SRMKFLSVVPHVIVFSNELPD-----------MNKMS
M9LTI9          YVNYGLLESIKDRMVLSTKYE--SRMKILAQNTHVIVFSNEQPN-----------YEKMT
914343032       FLQYNVLEQIKDRLIFSPKYS--STLKILTSNVHCVVFSNEEPD-----------VSQLS
M9MJD1          FLPYT-------------------------------------------------------
C_2pa           ------------------------------------------------------------
A0A0B4UH75      QKTIQWMNLFLQGSPMNIRKK--GSQYMKMKNLPVIILSNYTLGDCYP-KARDDGRLETL
A0A075J175      HKKIQFLNAWADGQPLPLRQK--GSQLTKTDNLPLIIVSNYSIEEVYR----PGVGRDAL
A0A0B4UI50      HKKIQFLNAWADGQPLPLRKK--GSQSVKTDNLPLIIVSNYTIEECYK----SGVGRDAL
P18921          RIDLQWMSDLTRSGALESGDE--------------------------------ACTSVG
A0A0H3XQH5      --WWEVGLTVPQ------QYMCNRSPSPSQTG-PSASTSVDQPD-----------PARRN
A0A0D5BUM7      TSWFKSLLGAQKDIILKGKYM--KDFRISGGI-PCIVLVNEDMD-----------WVVRM
C_869           KMDYQALEELKDGIWMAGKYK--GSTHIGGWP-TIIVLANRPPD-----------KTQLS


C_21            KRRVTKVHELDISSV------------------HAP-EDPEWDHF----------WQEPE
C_71            YRRFSKVMEYTGRNE------------------YVE-HDTNFETV----------GEGGA
C_5pa           MRRITHWIELDE-----------------------------------------------DGEQ
C_1388          FRRFTAVLDFTSPTDDKPYTVHMGVNASMRWFGYVK-CNGQWLTIEDAALQRSTKRKSPE
C_170           ARRISQIYDYSSGTL----VVRRELDSR-----NTF-WEREREHV----------ESDPA
Q1L2D4          FRRINRWLVWDA---------------------------TQFV-------------DAPE
Q06J75          FRRINRWLVWDG---------------------------LRFE-------------DAPE
D4N3Q0          FRRINKWMIWNA---------------------------TGFE-------------DAPD
Q0H9S9          FRRINKWLTWNA---------------------------IRFE-------------DAPD
Q9IG45          FRRINKWLVWNH---------------------------DKFE-------------DAPD
E9NWT8          FRRINKWLVWNH---------------------------DKFE-------------DAPD
Q06J78          YRRINVYKFYET---------------------------GEFK-------------DMPG
```

```
A7LI84          YRRINKYLVYNI---------------------------DKYE-------------PAQA
Q8AYV0          YRRINQYLILTP---------------------------DGYA-------------PAPE
F4YBZ7          MRRVNVYLVYDN-------------------------AGERFV-------------NLRE
G3LIS4          YRRITTLQFWKTAGEQ-----------------STEVPEGRFEA-----------VDPPC
B2CR69          YRRITSLVFWKNATEQ-----------------STE-EGGQFVT-----------LSPPC
C_351           LRRINITIFH--------------------------------------------------
G1D7G0          RRRFTEVVHFN-------------------------------------------------
A0A0C5IBG4      LRRLKITQFA-I------------------------------------------------
S4TE56          MRRLTITHFQ--------------------------------------------------
C_176           NDRYNIIEV---------------------------------------------------
922073311       ADRINIINV---------------------------------------------------
922073300       RDRFRVTHIRQV------------------------------------------------
922073297       HDRYHVTHIRQFPRADDN------------------------------------------
A0A0C5IB82      ADRFAVTHIRNI------------------------------------------------
S4TE35          DDRYNIKEL---------------------------------------------------
914343026       LDRYYIKEVN--------------------------------------------------
M9LTI9          TDRYKVTELN--------------------------------------------------
914343032       SDRLRVVRLDDS------------------------------------------------
M9MJD1          ------------------------------------------------------------
C_2pa           ------------------------------------------------------------
A0A0B4UH75      QARLDVIEVDSFIDF------------------YKD------------------------
A0A075J175      VDRFTQVFVDTAFVIDDP--------------EHVE------------------------
A0A0B4UI50      VDRFTQVFVDSLFSIDDP--------------LYTE------------------------
P18921          QQELERLLGPEVLEL------------------ITT-GSTQ-------------------
A0A0H3XQH5      GPDL--------------------------------------------------------
A0A0D5BUM7      ASTLKPWFDDNVLVY------------------YMS-ASDKFY-----------------
C_869           LNRWHIVRIPN-------------------------------------------------


C_21            LFIETPFFNQRTQ-----------------
C_71            NVSAIPYFEYEAPV---ETNARFIK-----
C_5pa           TIHKGPVISEDSSQ----------------
C_1388          SLSPVYASLYDEDRGGLAKKQKKLRRPGSK
C_170           SILAGGFGT--------------------T
Q1L2D4          VMKKYPINY---------------------
Q06J75          CMKKYPINY---------------------
D4N3Q0          CMKKYPINY---------------------
Q0H9S9          CMKKYPINY---------------------
Q9IG45          CMKKYPINY---------------------
E9NWT8          CMKKYPINY---------------------
Q06J78          HMLPHPINY---------------------
A7LI84          CTLPFPINY---------------------
Q8AYV0          FMLPFKINY---------------------
F4YBZ7          SAMYDPVTM------------RYCY-----
G3LIS4          SLFPYKINY---------------------
B2CR69          PEFPYEINY---------------------
C_351           ------------------------------
G1D7G0          ------------------------------
A0A0C5IBG4      ------------------------------
S4TE56          ------------------------------
C_176           ------------------------------
922073311       ------------------------------
922073300       ------------------------------
922073297       -------------------------GA-NG
A0A0C5IB82      ------------------------------
S4TE35          ------------------------------
914343026       ------------------------------
M9LTI9          ------------------------------
914343032       -----------------------------A
M9MJD1          ------------------------------
C_2pa           ------------------------------
A0A0B4UH75      ----------RSDI---------------L
A0A075J175      ------------------------------
A0A0B4UI50      ------------------------------
P18921          ------------------------------
A0A0H3XQH5      ------------------------------
A0A0D5BUM7      ------------------------------
C_869           ------------------------------
```
