# Supplementary material for: Virome Assembly and Annotation: A Surprise in the Namib Desert
Source: Front Microbiol. 2017 Jan 23;8:13. doi: 10.3389/fmicb.2017.00013 (PMC5253355; doi:10.3389/fmicb.2017.00013)
Supplement: Figure S6 — Multiple sequence alignment of circoviral capsid-like proteins (MSA conducted using MAFFT). [file Image6.PDF]

CLUSTAL W (1.8) multiple sequence alignment

```
A0A0C5I9R7      ---------------------------------------------------MSKRS--Y-
A0A0C5I9S3      ---------------------------------------------------MSKRGNPY-
S5SYF7          ------------------------------------------------MPGYNRRSQFR-
S5SYC3          ------------------------------------------------MPGYNRRSQFR-
S5SYG4          --------------------------------------------------MAYNRRTFE-
C_131           MLVHSITTNLRPASTPNLGVE-LVNFQESILKLNKNHLFFYNDLLNPQKKMPTRKRKRQ-
S5T672          MSIPAQTWNTLGFGTRATAAEALAGDALIAAGPVALGAAALGGIAYAGYKQMARRRPRS-
C_sub2          ---------------------------------------------------MYRKRKRS-
C_21            ----------------------MSLVTYPDVKNMSYGELIGHALQYAAENVPSRKRARS-
C_71            --------------------------------------------------MATRKRSRSG
C_130           --------------------------------------------------MATRKRGRSG
S5SYD5          ---------------------------------------------------MPRKYLKP-
C_2264          ----------------------------------------------MGMPYYPRRNYRR-
C_2936          --------------------------------------------------MAYKKMNYK-
A0A0B4UGX4      ------------------------------------------------MRGPTKRNYKG-
A0A0B4UGN4      ---------------------------------------------------MSRQVGQK-
A0A075IXZ7      ---------------------------------------------------MKRQRSAY-
A0A075IY03      ----------------------------------------------MSMKYLGKRKPTT-
P03606          ---------------------------------------------------MAKQQNNR-
O57031          ---------------------------------------------------MTKRQKRT-
P25879          ---------------------------------------------------MTKRQSKQ-
P29151          ----------------------------------------------MATQLTTRARRAT-
A0A0B4UI52      ---------------------------------------------------MAYPRGRR-
C_39            ----------------------------------------------------MKRRRQSG
C_172           ----------------------------------------------------MKRGAGAR
C_33pa          --------------------------------------------------MRQNRPYQQ-
A0A0K1RL58      ---------------------------------------------------MLKQRYKQ-


A0A0C5I9R7      -NFGTNPSQQYGVSRATKRRRNVNQYQ----NLPRGMNQRQIIRPYRLYTPRTPGGQVIA
A0A0C5I9S3      -NFGTNPSQQYGVSLASHRRGKDNRYQ----NLPRGMNQRPINRPYRLYTASIPGGQVIA
S5SYF7          SQSSSRPSKRWAESRASGARSQAVFTA----RARTAMARGRFAGHYR------KSGYYGR
S5SYC3          SQSSSRPSKRWAESRASGARSQAVFTA----RARTAMARGRFAGHYR------KSGYYGR
S5SYG4          --MVVNPRPYRKRGRSWNAPVGGVYKK-------RRIASAQKGYVR-------TTGYYGR
C_131           --------------RTSYGAPPAYMGR----R--RRFPKRQ-------------------
S5T672          -----FPGPYRPKKRGSYGPRSRPPSR----R--YRLPNLR------------IGGWLNR
C_sub2          ---------NTNNRRRTTSRPRKRYRPPRTMTKRRRMMNMR------------TGGLLDT
C_21            -----NSTVTTRMSGAGNVIGARYF-A----RPRRRYYNRR------------TGGFTGM
C_71            YDYGKYSQSYTKGRRRGDARPGAFRRP----R-GRSRRNTR------------TGGFLGI
C_130           YDYGKYSQSYTKGRRRGDARPGAFRRP----RSGGRRRNYR------------TGGYLGI
S5SYD5          ----------------------------------------------------VSRDYAYP
C_2264          ----------RPYRRRYTRRKTTGWQS----TAMKALTLAKR-----------VAGMLNV
C_2936          --------------------RTARAYA----PLLRRYANAQAMPGKAL--AKIAMGRANK
A0A0B4UGX4      ------SSRKIAAARAAVGRANAVLAS------RSSMSSGSYGPPA-------SRGFYGQ
A0A0B4UGN4      ----WKAPKQLQAPRNAFTNNNRNPKA-----------NGRSAPLA-------TRGFGDM
A0A075IXZ7      ------EIENRKQAARAKRRALVAARA----APMRAYANSQVPLR--------SGGY---
A0A075IY03      ------------WKPRSNPLTMMRAKA----RTSAFRMTPRAPLS--------TRGW--R
P03606          ---------------------RKSATM----RAVKRM-----------------------
O57031          ----------------QTTRSVRSLTT----RDVRNI-----------------------
P25879          ----------------SNRKSVAS-------QVRSI------------------------
P29151          ---------------RVSRKGSQPASK----QDVKQV----------------VKSILGQ
A0A0B4UI52      ---------TPLKRRATYGRGTRTFAR----TKFRRA-------------------YFNR
C_39            GSY-GYKKKRTNGAKRTYSRGQAYQFS----PKPMII-----------------------
C_172           DFYGGYKKTRTNGNRKKYSKGSAYQFN----PRPMVI-----------------------
C_33pa          ------KDKRPSASKRGRPAGYGDGAP----SASMMR--------------------LAQ
A0A0K1RL58      ---------TFTGKRATYRKKQRNYAP----KRNMQM-----------------------


A0A0C5I9R7      -------ERKYFDETLAAKIVIAAVTDFNDAEVDPAVRL---CLFAPTQGNDISNRESRS
A0A0C5I9S3      -------ERKYFDETLAAKIVIAAVTDFNDAEVDPAVRL---CLFAPTQGNDISNRESRS
S5SYF7          YNRDTGTELKFLDTSIDDA------------IVSTTLTVQTAGPNLIPQGTAEDERVGRK
S5SYC3          YNRDTGTELKFLDTSIDDA------------IVSTTLTVQTAGPNLIPQGTAEDERVGRK
S5SYG4          -FSGKPGEMKFFDTDKTGT------------TVAAAGTVLNSSLLLIPDGTTQSTRIGRK
C_131           QMKRLQPELKFLDVDLDDS-----VVAAGWSQAESGGAAV--DQLTIPEGTNESERIGRK
S5T672          -------ELKFKDVQRNVS----ISTTIAGSEIDPDGGVN--CLNAISQGDGEEQRDGRG
C_sub2          -------EIKFLDVNRSETALTAPTDCTGMEIQPSSGCTG--CLNAPVQGDGPSQRDGHK
C_21            -------ELKFVDSEETATVSSTPAG-----AEADNTTML--CLNGIAQGDGENQRDGRE
C_71            -------ELKFLDCAWNAVTINSSSDGAGGELQPSSGCTD--AISVPQQGDGESQRDGRK
C_130           -------ELKFLDCAWNGVTISSSTTGANGEMQPSSGCTD--CISVPAQGDGESQRDGRN
S5SYD5          GFARYKPELKHFDTAVDAT--------WGGSVITSPVLAS--SVLAIPEGAGENERQGRV
C_2264          -------EYKHKLTSFNAA-------------VDTSGNIT--QVTDIDQGDTSSTRGGNR
C_2936          ALKQLNSEKKYHDVTEADK-----------AIAKDTPEIY--VLNGIAQGDGEDQRVGFN
A0A0B4UGX4      YSLRGRAELKFVDATSTNA------------AVTTTWQGA--LINGIAQGADFNQRIGRK
A0A0B4UGN4      -GILKNREKKFFDRSTLGA----------PTGVSTTPVAY--LLHNPAQGADYNQRIGRK
A0A075IXZ7      --RFGSTEKKVFDLDTGTT------------NINTTGSVL--ALFVPTLGTDMTNRIGRK
A0A075IY03      PFISTNHELKVQDLASATY------------QVNTTGSIT--LLAVPITGADYNARIGRK
P03606          --INTHLEHKRFALINSGN------------TNATAGTVQN-LSNGIIQGDDINQRSGDQ
O57031          --IRGEAETKRFVLVNTLN------------LNSTAGTVLN-LSNGIIQGDDISQRAGDK
P25879          --VESMAEQKRFAFLTNTN------------TVTTAGTVIN-LSNNIVQGDDLVNRTGDQ
```

```
P29151          -----SLEHKRANLLLPPT------------VVNTTGNIYC-LTQFVIEGDGISQRTGRV
A0A0B4UI52      RRGVGRKEKKGCDVSFATA--------GIIASTSTNGGIY--LLNGIQEGVGSWNRIGRY
C_39            -VSTGRSELKGMDTDISIT------RGNVLATTNTNGAST--ILNLIRSGSGSWNRIGRK
C_172           -VESRRGELKGMDTDLSIN------QGNVVATTNTNDACI--ALNLIRAGTGSWNRIGRK
C_33pa          QAAMRARELKGLDTELSTG--------PVLATTTTNGNAF--VLNLIRPGTGSMNRIGRK
A0A0K1RL58      SIRARAGEKKLLDTDINTS--------QMLSTTGTNGGVN--CVNLIRSGNGYYNRIGRK


A0A0C5I9R7      CFVYSITVRGSIEMLGQGQQT--AID--NNQIARLVLVLDKQTNGTQ-MSSE---DLL--
A0A0C5I9S3      CFVYSITVRGSIEMLGQGQQT--AID--NNQIARLVLVLDKQTNGTQ-MSSE---DLL--
S5SYF7          VVVKSIHWRYRCLLPSSTSQN--D----TSEVIRVMLLLDKQCNGSY-PGAS---EIL--
S5SYC3          VVVKSIHWRYRCLLPSSTSQN--D----TSEVIRVMLLLDKQCNGSY-PGAS---EIL--
S5SYG4          ITVKHIFVHGHLILPATTTPG--D----TSDRVRIIMYLDKQCNGAA-AAVS---DIL--
C_131           VTITKIMGHYRVTMPSTSTPA--D----TSEYFRLAVVHDKQANGAV-PTLTGDAGIY--
S5T672          YKITSVHLRGYVLFAGQSGAG--A----TSDHMRIILLQDTQTNGTQ-FNAE---DVI--
C_sub2          IKCLSIFIQGYIAFPEATGDQ-----VREMPKVSIALVQDSQTNGVT-INSE---DIY--
C_21            VVIKQVMVRGIISLDTAASLA-------GANTCFIALVHDKQTNGAQ-FNAE---DVY--
C_71            YAIKSVWVSGTIDTTPKEDQG--DML--ETAGTFFALVLDTQANGST-VNSE---DVY-L
C_130           FIIRSIWVSGEVDWTTLTGQT--TFG--ELGNLFFALVLDKQANGST-IVSE---DVY-L
S5SYD5          ALIKKMEWKGILGNLVNTSPA---VGALPNRDFIFYIVLDRQCNGAA-ATQN---DIF--
C_2264          IKVKSLYLFGNVRMNASATQT----------QYRILIIQDKHGTGTA-PTVD---DII--
C_2936          VKATSLYWRINL---TANGAT-------NSNLCRILVVLDKQGDGSL-PSVT---DVL--
A0A0B4UGX4      AQMKSVLFNGNF-FPGTTAAE--NAS--QGVYLRVVIVYDSQPNSGTFPGGT---DFL--
A0A0B4UGN4      TVVKSIYIRGRMFVEPVGLTPLPADQWCPGQQARLIVFIDYQPNGAT-PALT---DLL--
A0A075IXZ7      AIIKSFYLRGYVRCENSLTPT--SPQAGSSQQLRIIVLIDMQPNGAL-PAIT---DIL--
A0A075IY03      VTLKSCFIRGYLRIEPGAATV--GVA--QAQQCRFMLVLDLQPNGAV-FAIT---DLL--
P03606          VRIVSHKLHVR--------GT--AIT--VSQTFRFIWFRDNMNRGTT-PTVL---EVL--
O57031          IRMTKQILRVR--------AT--AIT--NSQTFRFIWFKDNTNRGTT-PSVT---EVL--
P25879          IKTIHQTLLTR--------CT--GIT--NSQSFRFIWFRDNTNRGTT-PAVT---EVL--
P29151          INLEQMVLRYRRTLDTTSA---------NSGFLRYIVFLDTQNQGTL-PAIT---DVL--
A0A0B4UI52      MWNKSIELDLTLRYTSSYGAL--DTDITSGEWVRAVLVWDKQPNGGTIPTFD---TIFGQ
C_39            VKLKSVRLRGLAQHVYSNQAT--TSNI-LSNTLRMVVVWDKQPSGATLPTFD---KIFGR
C_172           VKCKSVRIKGKANFIYRVEST--TLDI-KGNFLRMVVIWDKQPSGAALPSFD---TVFGR
C_33pa          IKCKSLRLKGLLTYQYTPEAT--TLDR-NGNVLRMVVVWDKQPSGVL-PAFD---AIFGQ
A0A0K1RL58      VFLKSLRVYGSFTATYTQPAD--GDN--DGSVVRMVVVFDKQPNGGSLPTFD---TMFGH


A0A0C5I9R7      TTNSGESPMVY-AYQNTA--NFGRFQILKDQFIEFEPCNIGGVTGSFWQGGGRK------
A0A0C5I9S3      TTNSGESPMVY-AYQNTA--NFGRFQILKDQFIEFEPCNIGGVTGSFWQGGGRK------
S5SYF7          QTNNDWQ-----SFNNLS--NKDRFVTLYDKTIALNSMGNGGN-------GTTNESNEVR
S5SYC3          QTNNDWQ-----SFNNLS--NKDRFVTLYDKTIALNSMGNGGN-------GTTNESNEVR
S5SYG4          TATDVN------SFRNLS--ETGRFHILYDKVHSMNASSGTTA------GAGGDQFGEYM
C_131           EDNNFL------TFNNLS--NKSRFTVLYNEVIPMQNNSGSYD-------GTNDQFGETS
S5T672          DNSSGVNELQTVAFRNLE--NTNRFKILKDIVVHKPTTGLAGNQAN---PGDVESNSATM
C_sub2          QNDIGTARGNLCWKRTMS--NTSRFKVIRRKTVSFSNSQQNITSV-----GDIERNGQVV
C_21            EQPLASNPTG--LFRNLE--YTGRFRVLKTKQLNMNPMAAAGN-------GTTDSRATVV
C_71            NPSSGTLAMLPQPLRNLQ--NSKRFRILATKYVPPGGVVAGTD--------GTNTNSLNV
C_130           NPGTSGIAMLPHPLRNLQ--NSKRFSILDSKTIYAPELITAQD--------AAGTFVIAP
S5SYD5          TEVSGYHLHK--AFVQLH--NSRRFKILKKFIIRLHQGGISANDAN--------------
C_2264          ADASAMPYSA--STALQD--DMRRFNILYDKLYNISSSSNQLL-----------------
C_2936          TATNVD------AFRELK--NAYRYKILYDRRVKVAPEGQTGDLV---------------
A0A0B4UGX4      GANDPN------TPLNLN--NRDRFSILIDVRKQIGSYLFNGTPAL---TAGSPQNA---
A0A0B4UGN4      NSADPA------SQLNAD--NRDRFKVVKDKVFTFGPVCVSNSDLV--------SSERSY
A0A075IXZ7      KEQFPS------SQLNIN--NRDRFKILKDKTYALDTFIYNTTATQ-----AVGVAGRTL
A0A075IY03      NTADPT------SQLNLN--NRDRFRVLQDQEIAFDHFNYVTTATQ-----TSEAFGRTI
P03606          NTANFM------SQYNPITLQQKRFTILKDVTLNCSLTGESIKDRI-INLP---------
O57031          NSASFM------AQYNPVTLLQHRFTILKDVELDCSLSGESIKHLV-MTHG---------
P25879          DSASIT------SQYNPTTFQQKRFTVFQDFMLDTSIVGRVIVHRTAVDKK---------
P29151          SSLDVS------SGYEVLNAQQNRFKFLLDEVESLCASATNLSKAS--------------
A0A0B4UI52      TSQAGVESASVMDHLRYD--NMFRFKVLMDECINPTLTNAAVSTPINPASASLVNNTYF-
C_39            TEQDGTESTDFLDPINYD--NMGRFQVLADKLIDGKVSIVPNS-------GGSENDVFED
C_172           TEQDGTESSDILDAVKYD--NMGRFQILGEKVIDANIKHLPAS-------GGTTDEVISE
C_33pa          TAQDGSETSEFMDSLRYD--NTDRFRVLRDVVVTGSPQLDAS--------GGTVNETDVQ
A0A0K1RL58      TTQDGTESTNVLDPIKPD--NFGR------------------------------------


A0A0C5I9R7      ----MFKLKHTF-KEPIRVNFGSTNGGTVADIVDNSFHIICGTQNA-----SITTVLNYK
A0A0C5I9S3      ----MFKLKHTF-KEPTRVKPKSTNGGTVADIVDNSFHIICGTQNA-----SITTVLNYK
S5SYF7          H---CEEFHKKV-NIPINFD---STTGAVTEIASNNLVVALISE-------GGLPVFNSN
S5SYC3          H---CEEFHKKV-NIPINFD---STTGAVTEIASNNLVVALISE-------GGLPVFNSN
S5SYG4          R---GFSIKKAC-NYPIEYN---NTTGAIGEIRSNNXGIXAISA-------SGHCTATYK
C_131           Y---YREFYKDV-NIPIEYD-NSATTGAIGTIRSNNILVLYCTA-------SGLPSFAGT
S5T672          ----PIQMDVNF-KYPVNVL-CTGTGGTVSNLTDNSFHLMAISV-------NSGNSFRYI
C_sub2          RFQMSCSFPN---GLPVQFL-TGQTTADVANVVDNSFHIIAAATDI-----SVAPVIAYT
C_21            K---PFNFYHKL-NMPVRYT---GTGATVANITDNSLHLIIIG--------TGNMSFAWS
C_71            QNAMCVSLNW---KGEIICD-STGTTADVASAADNAIHLICYSGGL-----Y-SKKFTGK
C_130           GQRPCVSLNW---KGSIKVE-TSGTTADVANATDNAIHVLAYASSV-----QGTPVFQGK
S5SYD5          -----------------------GKGDYVHQSICFSVFLHFQGT--------------YY
C_2264          ----NLKYYKRV-DIPVIYR---GTSGT--DEGDNQFYVIQISNES-----TNTPIANWD
C_2936          ----TLKGYTNF-KYPVMVR-YNADTTSVSSITTNPIYLIAFSDALSS---GSPPVMDFI
A0A0B4UGX4      ----YWNKYKKC-NKETIFS---GTAATLGSISTGAMYIFFVGDF------NGVGMIDFY
A0A0B4UGN4      Q---DFKFFKKL-NLECINN--STSTNAIADIQSGALFAYFIGTNVAG---STDLVSVWT
```

```
A0A075IXZ7      Q---NFKCYKKM-NLEVIFN--SGNAGTIADTQTGALLIATIGSTASG---TADGNLVMS
A0A075IY03      Q---SFKKYKRL-NCEMIFN--AVNGGTIADIASGALYMVWIGSNAAGA--NTDANAVLS
P03606          -------------GQLVNYN---GATAVAASNGPGAIFMLQIGD-------SLVGLWDSS
O57031          -------------GTSCFYN---GTTAVASANGPGAIFLLVIGD-------SIVGTWDVG
P25879          -------------RRAIFYN---GAASVAASNGPGATFVLVIGS-------HATGQYDVT
P29151          ----TLTFNQ---KVQVHYG---GAADAATSNRRNAVFFLELSDKV-----ATGPQTRLG
A0A0B4UI52      ----RFHKYVKLRNMPTNFS-GTANPMTTANISTGALYLILRTQSA-----TLTELW---
C_39            F---AFDCYVKL-GRETIFE-GNSEPMTIADISSGGLYVYFRGLRNISDVSEWQINANSF
C_172           H---TFDHYIKI-GRDTIFE-GNSSPMTIADISTGGLYIYFRASRNDNNISQWSISGMTT
C_33pa          F---PFDEFIKLRGCETVYS-GETTPQTIADISSGGLYVFFRAFRDDATT-NWLVQSISF
A0A0K1RL58      --------------------------------SSRRLWI--------------------D

A0A0C5I9R7      SRVSFT---G-
A0A0C5I9S3      SRAPFT---G-
S5SYF7          MRIRFE---G-
S5SYC3          MRIRFE---G-
S5SYG4          ARVRYL---DQ
C_131           IRYRFM---DQ
S5T672          CRTRFV---G-
C_sub2          SRMRFV---G-
C_21            SRVRFV---G-
C_71            SRVRFV---G-
C_130           SRVRFI---G-
S5SYD5          HRL--------
C_2264          FRVRYL---DN
C_2936          SRFRYV---DN
A0A0B4UGX4      TRVRYT---DM
A0A0B4UGN4      SRTRYE---DM
A0A075IXZ7      SRIRFL---DP
A0A075IY03      TRVRYS---DS
P03606          YEAVYT---DA
O57031          YEAHYL---DL
P25879          AEIVYL---DM
P29151          VQLKFT---DA
A0A0B4UI52      ---------GH
C_39            ARLRYT---DY
C_172           SRLRYT---DY
C_33pa          ARLRYV---DV
A0A0K1RL58      MVIKFILKEDL
```
